# Transcription-protein dissociation reveals disrupted cellular stress pathways in the prefrontal cortex of depression and schizophrenia subjects

**DOI:** 10.1186/s43556-026-00514-4

**Published:** 2026-08-17

**Authors:** Cristina Ulecia-Morón, Álvaro G. Bris, Lucía Inglada-Pérez, Luis F. Callado, J. Javier Meana, David Martín-Hernández, Karina S. MacDowell, Elena Figuero, Javier R. Caso, Juan C. Leza

**Affiliations:** 1 Department of Pharmacology and Toxicology, Faculty of Medicine, University Complutense Madrid (UCM); Research Institute of Hospital 12 de Octubre (Imas12); University Institute of Research in Neurochemistry of the UCM (IUIN-UCM), Madrid, Spain; 2 Biomedical Research Network Centre in Mental Health, Institute of Health Carlos III (CIBERSAM, ISCIII), Madrid, Spain; 3 Department of Statistics and Operational Research, Faculty of Medicine, UCM, Madrid, Spain; 4 Department of Pharmacology, University of the Basque Country, UPV/EHU, Leioa, Bizkaia Spain; 5 BioBizkaia Health Research Institute, Barakaldo, Bizkaia Spain; 6 ETEP (Etiology and Therapy of Periodontal and Peri-implant Diseases) Research Group; Department of Dental Clinical Specialties, Faculty of Dentistry, UCM, Madrid, Spain

**Keywords:** Neuroinflammation, Autophagy, UPR, Depression, Schizophrenia, Postmortem brain tissue

## Abstract

Major depressive disorder (MDD) and schizophrenia (SCZ) are severe psychiatric disorders, the molecular mechanisms of which remain incompletely understood. Increasing evidence implicates neuroinflammatory signaling, autophagy dysregulation, and unfolded protein response (UPR) alterations in their pathophysiology. Here, we analyzed dorsolateral prefrontal cortex (DLPFC) samples from postmortem human brains of 28 MDD subjects, 28 SCZ subjects, and 28 matched controls. Gene expression levels of key inflammatory, autophagy, and UPR-related markers were assessed by RT-qPCR, while selected proteins were quantified by Western blot and ELISA. Logistic and linear regression models were applied to evaluate disease-associated alterations and the influence of sex, age, and cause of death. Transcriptional analyses revealed pathway-specific alterations in both disorders, with IRE1α emerging as the most consistently upregulated marker across MDD and SCZ. Sex-stratified analyses indicated that risk-associated transcriptional changes were more prominent in men with MDD, whereas women with SCZ showed broader transcriptional alterations. Age-related effects were mainly detected at the mRNA level, particularly in autophagy-related genes. In contrast, protein analyses showed a generalized downregulation of several inflammatory (AIM2, NLRP3), autophagy (ATG7, mTOR, RAB5A), and UPR-related (IRE1α) proteins in both disorders. In SCZ subjects who died by suicide, increased *IL18*, *CASPASE-5*, and *IRE1α* transcription, together with increased CASPASE-8 protein levels, suggested enhanced inflammatory and stress-related signaling. Overall, these findings reveal a marked transcription-protein dissociation in key cellular stress pathways in the DLPFC of MDD and SCZ subjects, supporting multilayer regulation of inflammatory, autophagy-related, and UPR responses in the psychiatric brain.

**Supplementary Information:**

The online version contains supplementary material available at 10.1186/s43556-026-00514-4.

## Introduction

Psychiatric disorders such as major depressive disorder (MDD) and schizophrenia (SCZ) form a major burden on global health, posing difficult challenges for diagnosis and treatment [1]. The underlying biological mechanisms driving these disorders remain poorly understood, and there is a persistent lack of robust biomarkers to guide diagnosis, prognosis, and treatment decisions. This scarcity of reliable biomarkers limits the development of targeted therapies and hinders efforts to identify individuals at risk at early stages of disease [2].

Recent research has progressively focused on the role of inflammatory processes, autophagy, and the unfolded protein response (UPR) in the pathophysiology of psychiatric disorders. Accumulating evidence points to the presence of neuroinflammation, altered cytokine profiles, and immune cell activation in both MDD and SCZ [3–6]. Autophagy, a lysosome-dependent cellular recycling system, contributes to the removal of pro-inflammatory substances such as protein aggregates and dysfunctional organelles, thereby functioning as a key homeostatic and anti-inflammatory mechanism [7]. Accordingly, dysregulation of autophagy has been proposed to contribute to the pathophysiology of psychiatric disorders [8–11]. Likewise, alterations in UPR signaling have been associated with psychiatric conditions, suggesting that disturbances in protein folding and cellular stress responses may play an important role in disease pathology [12–14].

Postmortem human brain tissue from individuals diagnosed with psychiatric conditions provides a unique and irreplaceable resource for investigating these molecular pathways. Such samples offer direct access to the biochemical and cellular landscape of the human brain, depicting the complexity of disease-related processes that cannot be fully recapitulated in experimental models. Consequently, the study of human brain tissue remains essential for closing the gap between research results and clinically relevant mechanisms underlying psychiatric illness.

In this context, the present study investigates whether key genes and proteins involved in neuroinflammatory signaling, autophagy, and the UPR are altered in the postmortem dorsolateral prefrontal cortex (DLPFC) of individuals with MDD and SCZ compared to appropriate control subjects. In addition, we examine the influence of key biological and clinical variables, including sex, age, and cause of death, on the modulation of these molecular pathways. By characterizing disease-associated alterations directly in the human brain, this work aims to provide novel insights that may contribute to the identification of novel molecular targets and support the development of more precise therapeutic strategies for psychiatric disorders.

## Results

### Altered IRE1α transcription is associated with increased risk of depression and schizophrenia, whereas RAB5 shows a protective association

To assess disease-related mRNA alterations in inflammatory, autophagy-related, and UPR pathways, selected markers from each molecular route were analyzed by logistic regression (Table 1). In MDD, altered *NLRP3, IRE1α*, *ATG7*, and *RAB7* transcription was associated with a higher disease risk, with *ATG16L1* showing a marginal association. In SCZ, *AIM2* and *IRE1α* expression changes were associated with higher disease odds, whereas *RAB5* transcription suggested a protective association. *GALECTIN 3*, *LC3α*, and *CYSTATIN C* showed marginal changes in SCZ. Thus, *IRE1α,* a key UPR-related ER stress mediator, was the only transcript associated with both MDD and SCZ.

**Table 1:** Logistic regression model of disease-associated gene and protein expression changes in inflammatory, autophagy, and UPR pathways in the prefrontal cortex of subjects with MDD and SCZ, compared to matched controls

**GENES/PROTEINS**	**MDD**			**SCZ**		
	***OR***	**CI 95%**	***p* value**	***OR***	**CI 95%**	***p* value**
**GENES – INFLAMMATION **						
***AIM2***	1.062	0.986-1.144	0.113	1.094	1.010-1.185	**0.027**
***ASC***	1.027	0.950-1.110	0.509	1.030	0.958-1.107	0.419
***BID***	1.027	0.928-1.136	0.607	1.027	0.973-1.106	0.265
***CASPASE 1***	0.995	0.960-1.030	0.761	0.995	0.973-1.019	0.701
***CASPASE 2***	1.133	0.893-1.438	0.303	1.133	0.945-1.302	0.204
***CASPASE 4***	0.997	0.988-1.007	0.546	0.998	0.992-1.004	0.499
***CASPASE 5***	1.003	0.997-1.009	0.373	1.006	0.997-1.014	0.179
***CASPASE 8***	1.044	0.987-1.104	0.133	1.044	0.974-1.101	0.261
***IL1β***	1.001	0.999-1.003	0.282	1.000	0.996-1.003	0.858
***IL18***	1.002	0.978-1.027	0.869	1.007	0.984-1.029	0.570
***NALP1***	1.004	0.994-1.013	0.486	1.007	0.993-1.021	0.322
***NLRC4***	0.998	0.993-1.004	0.501	0.997	0.988-1.006	0.529
***NLRP3***	1.018	1.001-1.036	**0.039**	1.009	0.996-1.023	0.179
***NRF2***	1.000	0.998-1.002	0.688	1.002	0.999-1.004	0.150
***TXNIP***	1.004	0.990-1.018	0.619	1.009	0.997-1.022	0.136
**GENES – AUTOPHAGY**						
***ATG3***	0.9944	0.9729-1.0163	0.6117	0.9965	0.9752-1.0181	0.7469
***ATG7***	1.0456	1.0177-1.0743	**0.0012**	1.0147	0.9952-1.0345	0.1414
***ATG16L1***	1.2387	0.9913-1.5479	**0.0597**	0.9547	0.7765-1.1739	0.6604
***BECLIN1***	1.0355	0.9408-1.1398	0.4757	0.9515	0.8725-1.0378	0.2617
***CYSTATIN C***	1.0002	0.9990-1.0014	0.7626	1.0016	0.9999-1.0033	0.0645
***GALECTIN 3***	1.0100	0.9931-1.0271	0.2486	1.0179	0.9998-1.0363	0.0526
***GALECTIN 9***	1.0712	0.9741-1.1779	0.1562	1.0030	0.9326-1.0787	0.9357
***LAMP2A***	0.9962	0.9856-1.0070	0.4878	0.9974	0.9886-1.0063	0.5665
***LC3α***	1.0214	0.9855-1.0586	0.2455	0.9618	0.9234-1.0017	0.0606
***mTOR***	1.0314	0.9821-1.0831	0.2157	1.0297	0.9774-1.0848	0.2713
***RAB5***	0.9989	0.9652-1.0338	0.9513	0.9560	0.9174-0.9964	**0.0329**
***RAB7***	1.0103	1.0001-1.0206	**0.0483**	1.0012	0.9926-1.0099	0.7834
***SQSTM1***	1.0067	0.9831-1.0310	0.5796	1.0106	0.9859-1.0359	0.4051
***ULK1***	1.0492	0.9564-1.1511	0.3093	1.0034	0.9161-1.0989	0.9423
**GENES – UPR**						
***ATF4***	0.997	0.979-1.015	0.763	1.000	0.993-1.007	0.908
***ATF6***	0.998	0.994-1.003	0.513	0.999	0.994-1.003	0.546
***BDNF***	0.998	0.993-1.003	0.474	0.984	0.967-1.002	**0.079**
***BiP***	0.994	0.975-1.012	0.503	0.981	0.958-1.005	0.117
***CHOP***	0.976	0.932-1.023	0.310	0.992	0.953-1.033	0.705
***IF2α***	1.000	0.999-1.001	0.697	1.001	0.999-1.002	0.272
***IRE1α / ERN1***	1.072	1.031-1.115	**0.001**	1.055	1.024-1.087	**0.0005**
***PERK***	1.005	0.983-1.027	0.663	0.995	0.977-1.012	0.558
***XBP1***	1.000	0.993-1.008	0.949	1.001	0.996-1.005	0.758
**PROTEINS – INFLAMMATION**						
**AIM2**	0.942	0.907-0.978	**0.0016**	0.959	0.933-0.986	**0.003**
**CASPASE 1**	0.996	0.957-1.037	0.852	0.999	0.963-1.037	0.975
**CASPASE 5**	0.995	0.989-1.00	0.062	0.987	0.567-1.495	**0.001**
**CASPASE 8**	0.982	0.651-1.482	0.932	0.921	0.98-0.995	0.738
**IL1β**	0.926	0.584-1.468	0.744	1.207	0.679-2.146	0.521
**IL18**	1.004	0.982-1.026	0.713	1.024	0.996-1.053	0.092
**NLRC4**	0.977	0.958-0.996	**0.018**	0.992	0.980-1.005	0.221
**NLRP3**	0.949	0.919-0.980	**0.006**	0.907	0.859-0.958	**0.0005**
**PROTEINS – AUTOPHAGY**						
**ATG7**	0.930	0.893-0.969	**0.0006**	0.881	0.824-0.942	**0.0002**
**mTOR**	0.940	0.907-0.975	**0.00007**	0.948	0.921-0.976	**0.0004**
**RAB5A**	0.937	0.903-0.972	**0.0005**	0.926	0.889-0.965	**0.0003**
**RAB7**	0.979	0.957-1.002	0.074	0.971	0.945-0.997	**0.027**
**PROTEINS – UPR**						
**IRE1α**	0.917	0.878-0.957	**0.00007**	0.707	0.500-1.001	**0.05**
**PROTEASOME 26S**	0.985	0.955-1.016	0.345	1.007	0.977-1.037	0.648

Gene expression changes were analyzed for all selected inflammatory, autophagy, and UPR markers. Protein-level analyses were performed only for markers showing relevant disease-associated alterations across the overall analyses and/or stronger biological consistency. This table shows the odds ratio (OR), confidence level of 95% (CI 95%), and *p *value predicted by the model for MDD and SCZ prefrontal cortex preparations. Significant results are shown in bold numbers. Underlined values indicate trends toward statistical significance

### Reduced inflammatory, autophagy-related, and IRE1α protein levels are associated with lower odds of depression and schizophrenia

Based on the transcriptional results, protein expression was evaluated for markers showing consistent changes. Caspases and interleukins were additionally measured to better characterize inflammatory signaling (Table 1). AIM2 and NLRP3 protein levels were associated with lower odds of both MDD and SCZ. NLRC4 was associated with a reduced risk of MDD, whereas CASPASE-5 was associated with lower odds of SCZ. CASPASE-1, CASPASE-8, IL1β, and IL18 exhibited no significant associations, although IL18 displayed a marginal association in SCZ. Regarding autophagy-related proteins, ATG7, mTOR, and RAB5A were associated with a reduced risk of both disorders, while RAB7 was associated with lower odds only in SCZ. IRE1α showed a lower-risk association with MDD and a borderline association with SCZ.

**Fig. 1:**
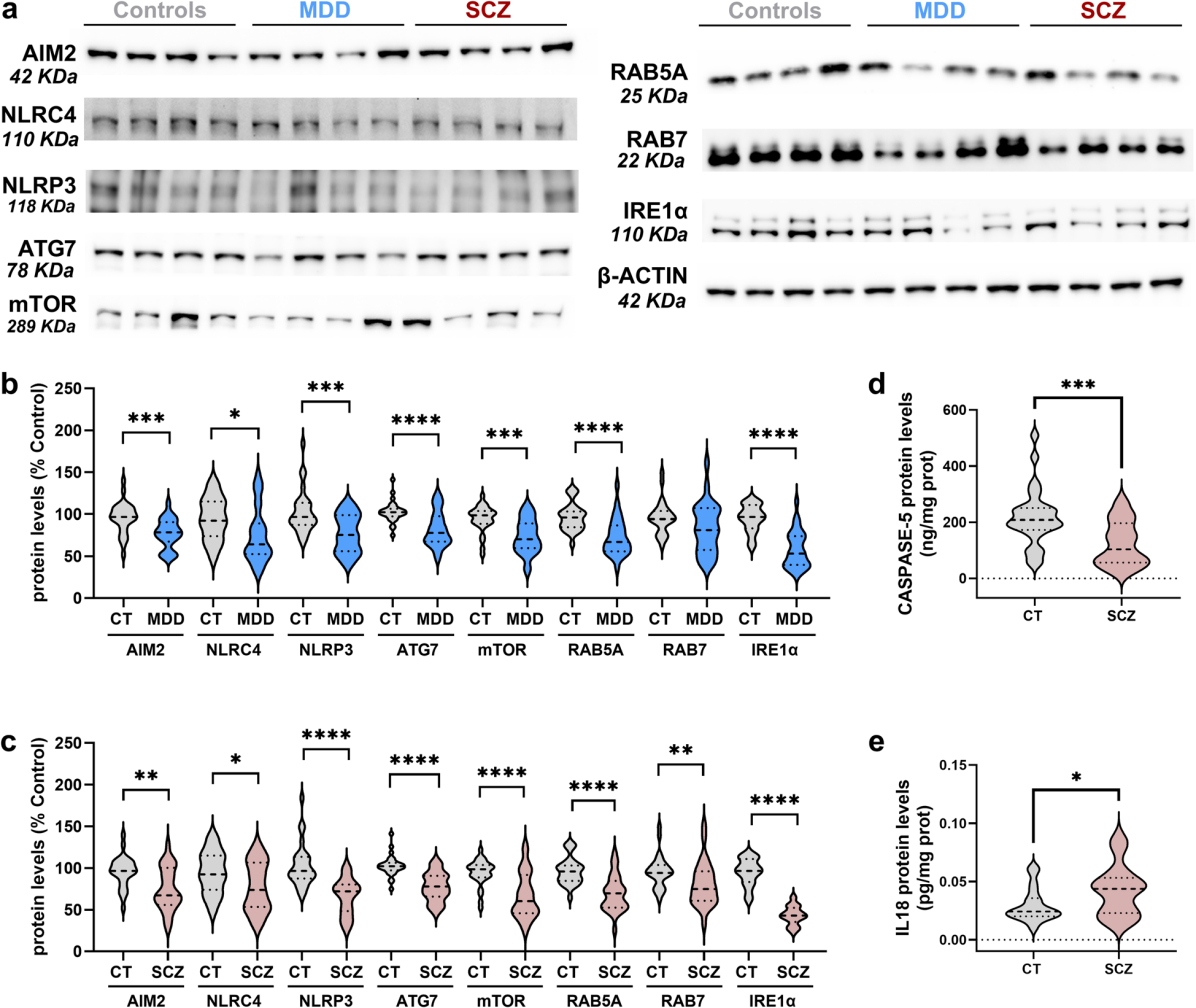
Protein expression alterations in inflammatory, autophagy, and UPR molecular markers in MDD and SCZ subjects compared to controls. Genes showing consistent transcriptional alterations were selected for protein-level analysis (**a**; Table 1). Protein levels were individually compared using the Student’s *t*-test (Table S1), and only statistically significant changes are exhibited (**b**-**e**). Western blot analyses showed protein alterations in the MDD cohort (b; blue) and SCZ cohort (**c**; red). CASPASE-5 (**d**) and IL18 (**e**) protein levels in SCZ subjects were quantified by ELISA. Representative Western blot bands correspond to the same individuals across all displayed proteins. Violin plots depict the kernel density estimation of data distribution, with the width of the shaded region reflecting the relative frequency of observations. Horizontal dashed lines indicate the mean, whereas dotted lines indicate the first and third quartiles (interquartile range). This graphical representation was chosen to facilitate visualization of the data distribution given the relatively large number of observations per group (*n* = 28). A *p < 0.05 was considered statistically significant (**p < 0.01, ***p < 0.001, ****p < 0.0001)

Individual group comparisons supported these results (Fig. 1; Table S1). AIM2, NLRC4, NLRP3, ATG7, mTOR, RAB5A, and IRE1α were all reduced at the protein level (Fig. 1a-c). RAB7 and CASPASE-5 were downregulated only in SCZ (Fig. 1d), whereas IL18 was induced in SCZ (Fig. 1e). Overall, reduced protein abundance in inflammatory, autophagy-related, and UPR pathways was associated with a lower risk of MDD and SCZ.

### Sex-dependent molecular signatures distinguish depression and schizophrenia at gene and protein levels

Sex-stratified analyses showed distinct molecular patterns in MDD and SCZ (Table 2). At the transcriptional level, most risk-associated changes were observed in MDD men, including *NLRP3, IRE1α, IL1β, ATG7,* and *RAB7*, with *ATG16L1* showing a marginal association. In SCZ, *RAB5* alterations in women were associated with lower disease odds, whereas *IRE1α* changes in men were linked to increased risk. *ATF4*, *BDNF*, and *XBP1* showed marginal protective associations in SCZ women. Complementary group comparisons confirmed higher *ATG7*, *ATG16L1, RAB7,* and *IRE1α* expression in MDD men, while SCZ women showed broader transcriptional alterations, mainly involving *ATF4*, *BDNF*, *RAB5*, and *XBP1* (Fig.S1; Table S2).

**Table 2:** Sex differences in the transcriptional and protein-level alterations of inflammatory, autophagy, and UPR markers in the DLPFC of subjects with MDD and SCZ compared to controls, using a logistic regression model

**GENES/PROTEINS**	**MDD Men**			**MDD Women**			**SCZ Men**			**SCZ Women**		
	***OR***	**CI 95%**	***p* value**	***OR***	**CI 95%**	***p* value**	***OR***	**CI 95%**	***p* value**	***OR***	**CI 95%**	***p* value**
**GENES – INFLAMMATION**												
***AIM2***	1.074	0.979-1.179	0.132	1.044	0.979-1.179	0.416	1.075	0.979-1.182	0.130	1.181	0.980-1.423	0.081
***ASC***	1.045	0.957-1.141	0.330	0.865	0.957-1.141	0.308	1.063	0.968-1.168	0.199	0.895	0.730-1.098	0.286
***BID***	1.147	0.865-1.520	0.341	1.010	0.865-1.520	0.859	1.044	0.886-1.231	0.604	1.030	0.970-1.093	0.336
***CASPASE 1***	0.999	0.943-1.058	0.960	0.978	0.943-1.058	0.784	1.015	0.956-1.076	0.630	0.922	0.792-1.074	0.297
***CASPASE 2***	2.068	0.879-4.869	0.096	1.047	0.879-4.869	0.715	1.207	0.838-1.738	0.312	1.068	0.927-1.230	0.363
***CASPASE 4***	1.011	0.964-1.061	0.642	0.996	0.964-1.061	0.655	1.034	0.984-1.086	0.189	0.996	0.983-1.010	0.610
***CASPASE 5***	1.002	0.997-1.008	0.400	1.014	0.997-1.008	0.411	1.005	0.997-1.014	0.215	1.018	0.980-1.059	0.358
***CASPASE 8***	1.150	0.913-1.448	0.235	1.025	0.913-1.448	0.346	1.150	0.913-1.448	0.235	1.021	0.962-1.083	0.496
***IL1β***	1.001	0.998-1.003	0.633	1.007	0.998-1.003	0.318	0.000	0.000-0.000	0.976	0.952	0.894-1.014	0.125
***IL18***	1.007	0.980-1.035	0.619	0.972	0.980-1.035	0.443	1.015	0.988-1.044	0.278	0.963	0.900-1.031	0.278
***NALP1***	1.004	0.993-1.014	0.490	1.007	0.993-1.014	0.790	1.007	0.991-1.023	0.389	1.009	0.977-1.043	0.577
***NLRC4***	1.014	0.966-1.064	0.586	0.998	0.966-1.064	0.488	0.999	0.961-1.038	0.940	0.997	0.987-1.007	0.546
***NLRP3***	1.022	1.000-1.044	**0.049**	1.012	1.000-1.044	0.491	1.011	0.994-1.027	0.198	1.006	0.981-1.032	0.646
***NRF2***	1.000	0.998-1.002	0.793	1.002	0.998-1.002	0.563	1.001	0.998-1.003	0.478	1.004	0.999-1.008	0.119
***TXNIP***	1.014	0.985-1.045	0.345	0.999	0.985-1.045	0.942	1.008	0.978-1.040	0.601	1.008	0.995-1.022	0.209
**GENES – AUTOPHAGY**												
***ATG3***	0.997	0.973-1.021	0.790	0.982	0.973-1.021	0.516	0.995	0.968-1.022	0.711	0.999	0.964-1.036	0.960
***ATG7***	1.050	1.014-1.087	**0.006**	1.040	1.014-1.087	0.088	1.010	0.987-1.034	0.395	1.031	0.988-1.077	0.157
***ATG16L1***	1.313	0.976-1.768	0.072	1.141	0.976-1.768	0.436	0.923	0.708-1.202	0.551	0.998	0.697-1.428	0.989
***BECLIN1***	1.104	0.965-1.264	0.149	0.951	0.965-1.264	0.521	0.998	0.896-1.112	0.975	0.873	0.744-1.025	0.096
***CYSTATIN C***	1.000	0.999-1.001	0.764	1.000	0.999-1.001	0.952	1.001	0.999-1.003	0.192	1.002	0.999-1.006	0.158
***GALECTIN 3***	1.011	0.992-1.030	0.254	1.004	0.992-1.030	0.855	1.020	0.994-1.046	0.134	1.018	0.992-1.046	0.183
***GALECTIN 9***	1.102	0.974-1.247	0.122	1.018	0.974-1.247	0.826	1.018	0.937-1.107	0.668	0.952	0.815-1.112	0.537
***LAMP2A***	0.995	0.983-1.007	0.408	1.005	0.983-1.007	0.743	0.996	0.986-1.006	0.467	1.002	0.982-1.022	0.832
***LC3α***	1.045	0.995-1.098	0.081	0.983	0.995-1.098	0.578	0.974	0.926-1.025	0.314	0.939	0.872-1.010	0.090
***mTOR***	1.040	0.979-1.105	0.208	1.015	0.979-1.105	0.744	1.041	0.967-1.120	0.284	1.019	0.944-1.101	0.621
***RAB5***	1.016	0.973-1.060	0.472	0.960	0.973-1.060	0.244	0.976	0.933-1.020	0.278	0.919	0.850-0.993	**0.033**
***RAB7***	1.024	1.006-1.043	**0.009**	0.992	1.006-1.043	0.386	1.008	0.995-1.021	0.257	0.993	0.979-1.008	0.356
***SQSTM1***	1.002	0.977-1.029	0.855	1.024	0.977-1.029	0.397	1.001	0.973-1.029	0.950	1.036	0.984-1.090	0.177
***ULK1***	1.049	0.948-1.161	0.352	1.049	0.948-1.161	0.681	0.980	0.878-1.095	0.726	1.062	0.891-1.266	0.502
**GENES – UPR**												
***ATF4***	1.006	0.982-1.029	0.640	0.983	0.982-1.029	0.300	1.004	0.993-1.015	0.461	0.948	0.896-1.003	0.065
***ATF6***	1.003	0.991-1.015	0.601	0.997	0.991-1.015	0.472	1.005	0.993-1.017	0.395	0.996	0.982-1.010	0.530
***BDNF***	1.005	0.987-1.024	0.594	0.997	0.987-1.024	0.526	0.992	0.972-1.011	0.398	0.957	0.915-1.000	0.052
***BiP***	0.991	0.969-1.013	0.432	1.000	0.969-1.013	0.980	0.988	0.963-1.012	0.324	0.941	0.875-1.011	0.095
***CHOP***	0.979	0.932-1.029	0.404	0.960	0.932-1.029	0.492	0.989	0.943-1.038	0.661	1.000	0.922-1.085	0.993
***IF2α***	1.000	0.999-1.002	0.790	1.001	0.999-1.002	0.712	1.001	0.999-1.002	0.462	1.003	0.999-1.007	0.211
***IRE1α / ERN1***	1.077	1.025-1.131	**0.003**	1.074	1.025-1.131	0.065	1.051	1.017-1.086	**0.003**	1.080	0.997-1.170	0.060
***PERK***	1.006	0.982-1.030	0.641	1.000	0.982-1.030	0.986	1.002	0.983-1.021	0.876	0.952	0.898-1.010	0.105
***XBP1***	1.001	0.992-1.009	0.882	0.999	0.992-1.009	0.879	1.004	0.996-1.011	0.335	0.952	0.907-1.001	0.053
**PROTEINS – INFLAMMATION**												
**AIM2**	0.936	0.893-0.981	**0.006**	0.954	0.895-1.017	0.152	0.959	0.928-0.990	**0.010**	0.960	0.908-1.014	0.145
**CASPASE 1**	1.001	0.959-1.046	0.954	0.952	0.836-1.084	0.455	0.990	0.948-1.033	0.640	1.043	0.952-1.143	0.363
**CASPASE 5**	0.995	0.989-1.002	0.150	0.992	0.981-1.003	0.171	0.985	0.975-0.996	**0.005**	0.990	0.978-1.003	0.136
**CASPASE 8**	1.200	0.715-2.015	0.491	0.586	0.243-1.414	0.234	0.932	0.538-1.614	0.802	0.876	0.304-2.521	0.805
**IL1β**	0.085	0.008-0.884	**0.039**	1.101	0.671-1.808	0.703	1.972	0.332-1.549	0.451	1.136	0.626-2.064	0.675
**IL18**	0.983	0.926-1.043	0.562	1.008	0.983-1.034	0.525	1.046	0.977-1.118	0.195	1.019	0.989-1.05	0.208
**NLRC4**	0.981	0.96-1.003	0.087	0.965	0.926-1.005	0.089	0.994	0.982-1.007	0.364	0.980	0.95-1.011	0.205
**NLRP3**	0.949	0.914-0.986	**0.007**	0.942	0.883-1.005	0.071	0.910	0.853-0.970	**0.004**	0.887	0.78-1.008	0.067
**PROTEINS – AUTOPHAGY**												
**ATG7**	0.918	0.869-0.970	**0.002**	0.952	0.893-1.016	0.142	0.860	0.776-0.952	**0.004**	0.906	0.828-0.992	**0.032**
**mTOR**	0.933	0.891-0.977	**0.003**	0.953	0.899-1.01	0.104	0.943	0.908-0.98	**0.003**	0.955	0.912-1.00	**0.048**
**RAB5A**	0.937	0.896-0.979	**0.004**	0.928	0.861-0.999	**0.047**	0.927	0.885-0.972	**0.002**	0.915	0.833-1.005	0.062
**RAB7**	0.956	0.923-0.991	**0.014**	1.017	0.973-1.062	0.459	0.947	0.908-0.970	**0.011**	0.995	0.96-1.031	0.778
**PROTEINS – UPR**												
**IRE1α**	0.914	0.866-0.964	**0.0009**	0.922	0.855-0.995	**0.036**	0.842	0.402-0.929	**1,14E-10**	0.860	0.257-0.951	**0.00008**
**PROTEASOME 26S**	0.763	0.57-1.022	0.070	0.996	0.968-1.024	0.764	1.049	0.964-1.141	0.265	1.001	0.97-1.032	0.965

Gene expression changes were analyzed for all selected inflammatory, autophagy, and UPR markers. Protein-level analyses were performed only for markers showing relevant disease-associated alterations across the overall analyses and/or stronger biological consistency. This table shows the odds ratio (OR), confidence level of 95% (CI 95%), and *p *value predicted by the model for MDD and SCZ prefrontal cortex preparations. Significant results are shown in bold numbers. Underlined values indicate trends toward statistical significance

At the protein level, associations were stronger and more consistent in men, particularly for inflammatory and autophagy-related markers. In MDD men, AIM2, NLRP3, IL1β, ATG7, mTOR, RAB5A, and RAB7 were associated with reduced disease risk, whereas only RAB5A was significant in women. IRE1α was associated with both sexes. In SCZ men, AIM2, CASPASE-5, NLRP3, ATG7, mTOR, RAB5A, and RAB7 were associated with reduced disease risk, while ATG7 and mTOR were linked to reduced risk in women (Table 2). Individual group comparisons confirmed reduced inflammatory and autophagy-related protein levels mainly in men, together with IRE1α downregulation in both sexes and disorders (Fig.S2; Table S2). Overall, risk-associated transcriptional changes predominated in MDD men, broader transcriptional alterations were observed in SCZ women, and protein-level associations were generally stronger in men.

### Age-related transcriptional regulation differs in depression and schizophrenia, whereas protein changes mainly involve inflammatory markers in schizophrenia

**Fig. 2:**
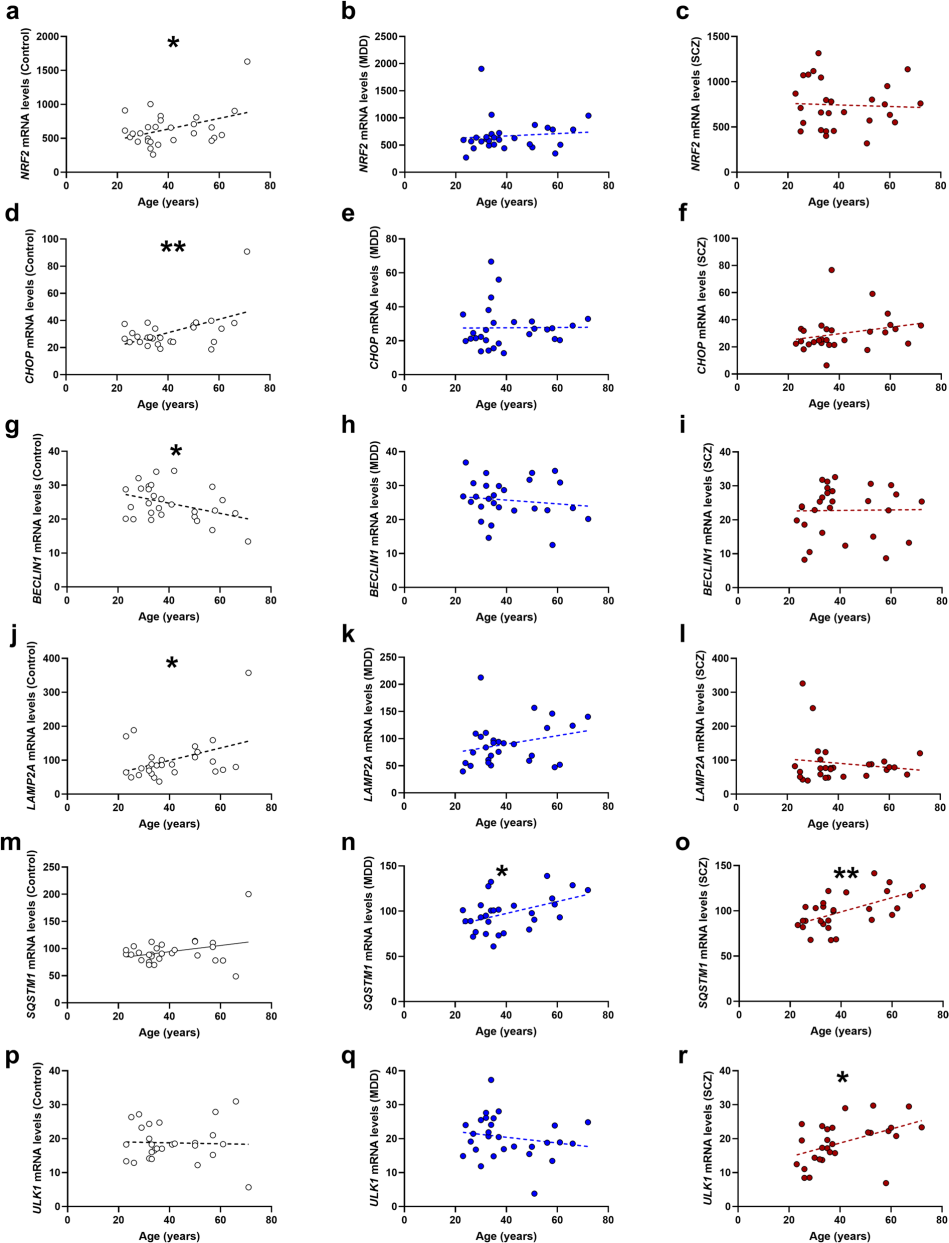
Significant age-related changes in the gene expression of inflammatory, autophagy-related, and UPR markers in controls, MDD and SCZ subjects. Gene expression levels of selected inflammatory, autophagy-related, and UPR markers showing significant age-associated changes are represented. All genes were analyzed according to age using linear regression (Table 3). In control individuals, *NRF2* and *CHOP* expression increased with age (**a**-**f**), whereas *BECLIN1* decreased and *LAMP2A* increased in older subjects (**g**-**l**). In contrast, these age-related patterns were not observed in MDD and SCZ patients. Regarding autophagy-related markers, *SQSTM1* transcriptional rate increased with age in both MDD and SCZ, but not in controls (**m**–**o**). *ULK1* transcription remained stable in controls and MDD patients but increased with age in SCZ (**p**-**r**). White dots exhibit controls (first column), blue dots represent MDD subjects (second column), and red dots illustrate SCZ patients (third column). A *p < 0.05 was considered statistically significant (**p < 0.01)

Age-related effects were analyzed using linear regression with age as a continuous predictor variable (Table 3). At the transcriptional level, controls showed age-associated changes in several UPR and autophagy markers: *NRF2, CHOP,* and *LAMP2A* expression increased with age, whereas *BECLIN1* decreased. However, these patterns were absent or reshaped in MDD and SCZ (Fig. 2a-l). In contrast, *SQSTM1* increased with age in both MDD and SCZ, but not in controls, while *ULK1* was selectively upregulated with age in SCZ (Fig. 2m-r). Other genes showing marginal age-associated effects are represented in Fig.S3.

**Table 3:** Age-associated differences in the transcriptional and translational rate of inflammatory, autophagy, and UPR markers in the DLPFC of subjects with MDD and SCZ compared to matched controls, using a linear regression model

**GENES/ PROTEINS**	**Coefficient (β)**			**SD**			***p*** **value**		
	**Control**	**MDD**	**SCZ**	**Control**	**MDD**	**SCZ**	**Control**	**MDD**	**SCZ**
**GENES – INFLAMMATION**									
***AIM2***	-0.053	-0.077	-0.075	0.075	0.204	0.198	0.491	0.708	0.708
***ASC***	-0.033	-0.051	-0.194	0.066	0.129	0.134	0.621	0.695	0.160
***BID***	-0.160	-0.075	-0.349	0.081	0.065	0.300	0.060	0.261	0.255
***CASPASE 1***	-4.260	-0.101	-0.272	3.278	0.156	0.152	0.205	0.521	0.085
***CASPASE 2***	-0.025	0.005	-0.115	0.038	0.033	0.164	0.523	0.889	0.492
***CASPASE 4***	-4.263	-0.104	-0.201	3.283	0.211	0.237	0.206	0.625	0.404
***CASPASE 5***	0.669	-1.599	-1.331	1.021	2.807	1.195	0.518	0.574	0.275
***CASPASE 8***	-0.103	-0.196	-0.096	0.092	0.279	0.186	0.275	0.489	0.610
***IL1β***	1.736	-6.591	-1.996	2.602	6.997	1.421	0.510	0.355	0.172
***IL18***	-0.142	-0.102	-0.657	0.237	0.377	0.393	0.555	0.790	0.107
***NALP1***	-0.709	-0.617	-0.883	0.570	1.005	0.534	0.225	0.545	0.110
***NLRC4***	-4.146	-0.094	0.270	3.154	0.373	0.265	0.200	0.803	0.318
***NLRP3***	-0.327	-0.965	-0.552	0.373	0.703	0.714	0.388	0.182	0.446
***NRF2***	7.858	2.142	-0.857	3.462	4.342	3.533	**0.032**	0.626	0.810
***TXNIP***	0.650	0.909	-2.629	0.512	0.541	3.765	0.216	0.105	0.491
**GENES – AUTOPHAGY**									
***ATG3***	0.548	0.261	-0.314	0.333	0.359	0.346	0.112	0.475	0.373
***ATG7***	0.188	-0.407	0.514	0.301	0.431	0.477	0.536	0.353	0.291
***ATG16L1***	0.007	-0.036	0.046	0.039	0.037	0.036	0.856	0.351	0.209
***BECLIN1***	-0.150	-0.056	0.008	0.068	0.086	0.100	**0.036**	0.520	0.939
***CYSTATIN C***	-0.922	-10.86	-4.527	4.195	8.041	8.393	0.828	0.188	0.594
***GALECTIN 3***	0.091	-0.392	0.705	0.324	0.650	0.831	0.780	0.552	0.404
***GALECTIN 9***	-0.121	-0.068	0.067	0.083	0.080	0.116	0.158	0.408	0.571
***LAMP2A***	1.835	0.774	-0.630	0.826	0.553	0.843	**0.035**	0.174	0.462
***LC3α***	-0.249	-0.236	0.069	0.191	0.244	0.206	0.204	0.342	0.738
***mTOR***	-0.027	-0.183	0.278	0.141	0.179	0.143	0.849	0.314	0.062
***RAB5***	-0.163	-0.483	0.190	0.166	0.250	0.276	0.333	0.065	0.497
***RAB7***	-0.637	-0.286	-0.650	0.770	0.851	0.939	0.416	0.740	0.495
***SQSTM1***	0.576	0.656	0.785	0.352	0.256	0.218	0.114	**0.016**	**0.001**
***ULK1***	-0.014	-0.085	0.206	0.079	0.090	0.076	0.860	0.350	**0.012**
**GENES – UPR**									
***ATF4***	-0.705	-0.525	-0.214	0.355	0.459	1.444	0.058	0.263	0.884
***ATF6***	-3.172	-0.430	0.117	2.498	0.907	0.915	0.215	0.639	0.899
***BDNF***	-5.074	-0.472	-0.090	3.059	0.599	0.501	0.109	0.438	0.859
***BiP***	-0.355	-0.445	-0.027	0.296	0.497	0.372	0.242	0.379	0.943
***CHOP***	0.495	0.010	0.239	0.164	0.176	0.178	**0.0056**	0.957	0.190
***IF2α***	7.674	13.601	7.172	4.553	7.027	7.393	0.104	0.064	0.341
***IRE1α / ERN1***	-0.184	-0.295	0.468	0.267	0.293	0.405	0.497	0.324	0.258
***PERK***	0.597	0.455	0.663	0.348	0.341	0.466	0.098	0.194	0.167
***XBP1***	-0.301	-0.786	-1.166	0.642	1.253	2.461	0.643	0.536	0.639
**PROTEINS – INFLAMMATION**									
**AIM2**	-0.168	-0.545	0.379	0.265	0.302	0.346	0.532	0.082	0.283
**CASPASE 1**	0.066	-0.017	0.126	0.241	0.120	0.153	0.787	0.891	0.420
**CASPASE 5**	0.413	-1.221	-1.234	1.456	1.600	1.086	0.779	0.452	0.266
**CASPASE 8**	-0.011	-0.016	-0.029	0.017	0.020	0.013	0.504	0.431	**0.038**
**IL1β**	-8.39E-06	9.47E-07	-1.78E-05	1.57E-05	1.79E-05	1.07E-05	0.597	0.958	0.108
**IL18**	-0.0004	-0.0001	-0.0007	0.00028	0.0004	0.0003	0.157	0.773	**0.013**
**NLRC4**	-0.377	0.077	-0.068	0.866	0.469	0.698	0.667	0.871	0.923
**NLRP3**	-0.214	0.052	0.530	0.369	0.326	0.279	0.567	0.873	0.068
**PROTEINS – AUTOPHAGY**									
**ATG7**	0.304	0.063	0.327	0.170	0.294	0.230	0.085	0.833	0.167
**mTOR**	-0.070	-0.151	0.009	0.225	0.294	0.373	0.756	0.612	0.981
**RAB5A**	0.150	-0.247	0.386	0.232	0.312	0.285	0.523	0.436	0.187
**RAB7**	-0.108	-0.424	0.259	0.274	0.418	0.359	0.696	0.320	0.478
**PROTEINS – UPR**									
**IRE1α**	-0.119	0.320	0.188	0.379	0.308	0.152	0.755	0.308	0.228
**PROTEASOME 26S**	-0.00011	5.21E-05	-0.00015	0.00032	0.00025	0.00019	0.739	0.833	0.438

This table shows the coefficient (β), standard deviation (SD), and *p *value predicted by the model for MDD and SCZ samples. Only those genes that underwent consistent changes across models were considered for protein quantification. Significant results are shown in bold numbers. Underlined values indicate trends toward statistical significance

Age-stratified comparisons using the median age of 35 years as cutoff showed more disease-related transcriptional changes in SCZ than in MDD (Fig.S4; Table S3). In MDD, *ATG7* and *IRE1α* were induced independently of age, particularly in younger subjects, while *RAB7* was upregulated only in older individuals. In SCZ, *AIM2* was increased in subjects over 35 years, whereas *ATF4* was reduced and *CYSTATIN C* increased in younger subjects. *IRE1α* was induced in both SCZ age groups, while *LC3α* and *NRF2* were altered in younger subjects with opposite patterns. Overall, age-related transcriptional regulation was more evident in control subjects for autophagy and UPR markers, and in SCZ for autophagy effectors.

Protein-level age-related effects were more limited. Linear regression analyses showed no significant associations in control subjects, whereas CASPASE-8 and IL1β decreased with age in SCZ, with NLRP3 showing a marginal association (Table 3). Individual age-stratified comparisons further showed that AIM2 was reduced in MDD independently of age and in younger SCZ subjects, CASPASE-5 was reduced in SCZ regardless of age, and IL18 increased in younger SCZ subjects (Fig.S5; Table S3). NLRP3 was reduced in both disorders and age groups, whereas NLRC4 decreased only in younger SCZ patients. mTOR, ATG7, RAB5A, and IRE1α were reduced in MDD and SCZ across both age groups, while RAB7 was selectively decreased in younger SCZ subjects. Together, these data indicate altered age-related transcriptional regulation in MDD and especially in SCZ, whereas age-dependent protein changes were restricted and mainly involved inflammatory markers in SCZ.

### Suicide-related IL18, CASPASE-5, SQSTM1, and IRE1α genes and CASPASE-8 protein alterations are present in schizophrenia subjects

**Fig. 3:**
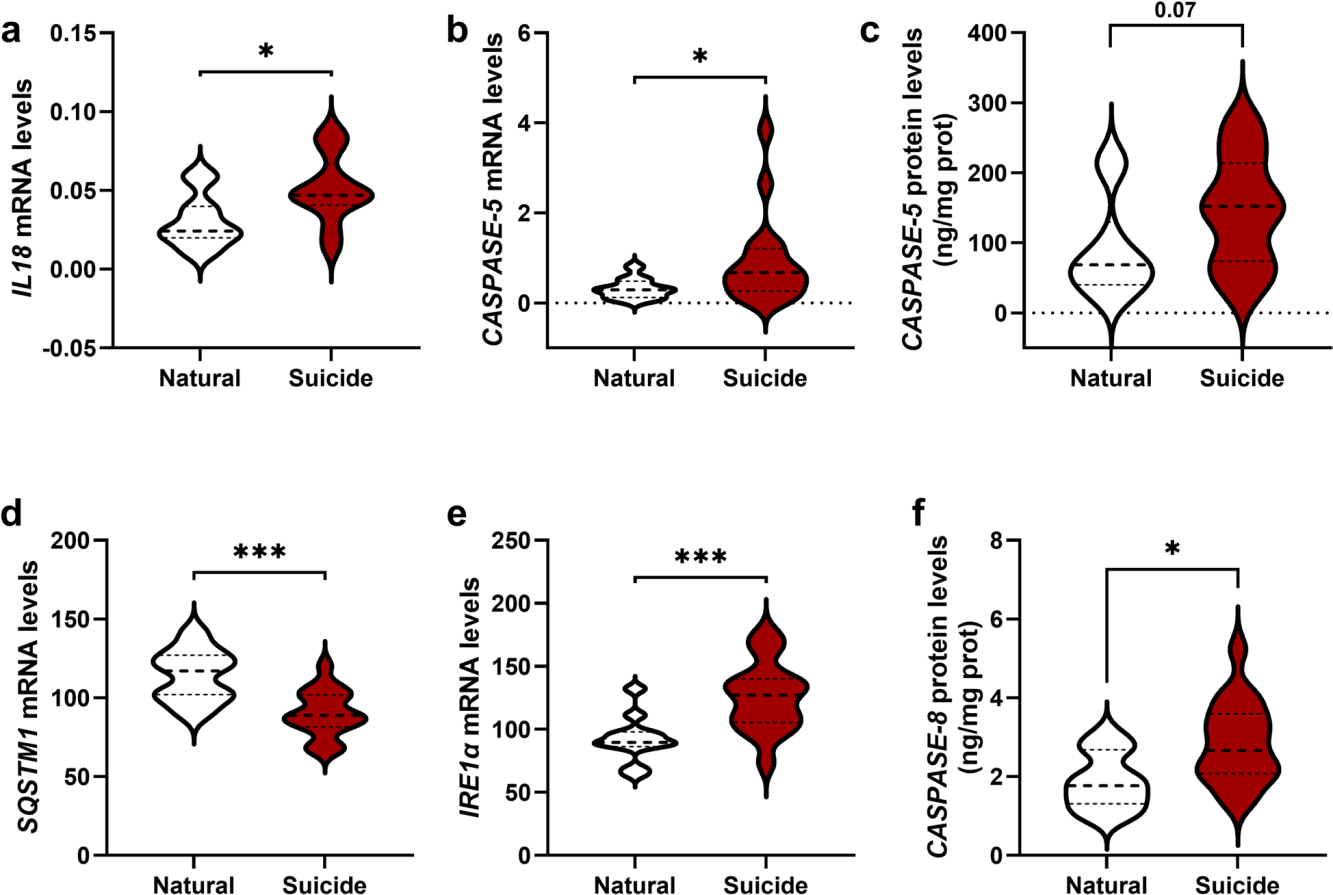
Suicide-associated inflammatory, autophagy, and UPR marker changes in SCZ subjects. *IL18* (**a**), *CASPASE-5* (**b**-**c**), *SQSTM1* (**d**), and *IRE1α* (**e**) gene expression, as well as CASPASE-8 protein levels (**f**) were altered in SCZ subjects who died by suicide (Table S4). CASPASE-5 protein levels showed a non-significant upward trend in suicidal subjects (**c**). White represents SCZ individuals who died from natural causes, and red indicates suicidal subjects. Violin plots show data distribution, with dashed lines representing the mean and dotted lines indicating the first and third quartiles (*n* = 11 and *n* = 17 in natural and suicidal groups, respectively). A *p < 0.05 was considered statistically significant (***p < 0.001)

To further explore molecular changes associated with cause of death in SCZ, subjects who died by suicide were compared with those who died of natural causes (Fig. 3; Table S4). *IL18* and *CASPASE-5* transcripts were upregulated in the SCZ suicide group (Fig. 3a-b), with CASPASE-5 protein showing a similar trend (Fig. 3c). *SQSTM1* expression was lower, whereas *IRE1α* transcription was higher in suicidal SCZ subjects (Fig. 3d-e). Additionally, CASPASE-8 protein concentrations were also increased in this group (Fig. 3f).

### Integrated gene-protein and pathway-level analyses reveal disease-dependent IRE1α separation and coordinated stress-response networks respectively

**Fig. 4:**
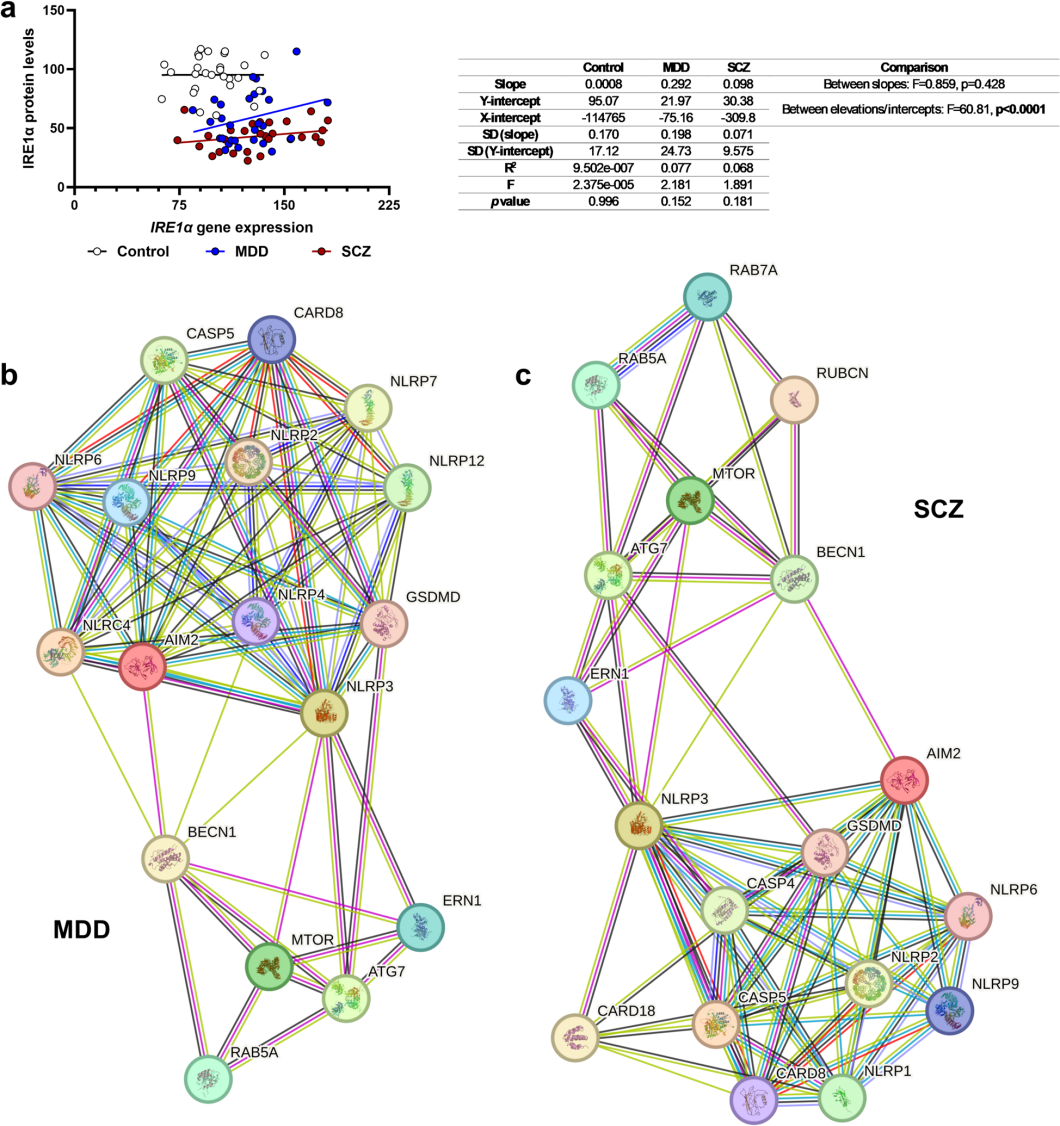
Integrated IRE1α gene-protein analysis and STRING-based pathway enrichment networks in MDD and SCZ. Relationship between IRE1α gene and protein levels in control, MDD, and SCZ subjects. Group differences in IRE1α protein levels were analyzed by ANCOVA, including disease status as the main factor. White dots represent controls, blue dots indicate MDD subjects, and red dots represent SCZ subjects (**a**). STRING-database protein–protein interaction networks generated from disease-associated markers in MDD (**b**) and SCZ (**c**; Table 4). Nodes represent proteins included in the network, and edges represent known or predicted functional associations derived from STRING evidence channels

To assess whether transcriptional changes were directly associated with protein abundance, Spearman correlations were performed for markers quantified at both molecular levels (Table S5). No significant correlations were detected, indicating that protein expression was not directly predicted by the corresponding transcript levels in these markers. Given the consistent involvement of IRE1α across the different analyses, its mRNA-protein relationship was further explored using ANCOVA-based comparisons across control, MDD, and SCZ (Fig. 4a). Slopes did not differ significantly, although elevations/intercepts did among groups, showing a clear group-dependent separation in IRE1α protein abundance. Controls displayed higher IRE1α protein levels, whereas MDD and especially SCZ subjects showed reduced protein abundance despite overlapping or increased transcriptional values.

To evaluate whether the disease-associated markers were functionally connected at pathway level, STRING protein–protein interaction networks were generated independently for MDD and SCZ (Fig. 4b-c). Functional analyses showed significant enrichment of NOD-like receptor signaling in both disorders, together with autophagy-related pathways (Table 4). Although MDD and SCZ shared the main enriched pathways, SCZ showed a distinct enrichment profile, with autophagy-related elements ranking higher and involving a larger vesicular trafficking/proteostasis module. Together, these analyses support disease-dependent IRE1α mRNA-protein separation and coordinated enrichment of inflammatory, autophagy-related, vesicular trafficking, and UPR pathways in both disorders.

**Table 4:** Functional enrichment analysis of STRING-expanded protein-protein interaction networks in MDD and SCZ

Term ID	Description	Observed gene count	Background gene count	Strength	Signal	FDR	Matching proteins in our network (labels)
**MDD**							
hsa04621	**NOD-like receptorsignaling pathway**	8	173	1.73	3.17	**3.29E-10**	NLRP6, NLRP3, AIM2, NLRP12, CASP5, NLRC4, GSDMD, CARD8
hsa05132	**Salmonella infection**	5	209	1.44	1.39	**0.00013**	RAB5A, NLRP3, CASP5, NLRC4, GSDMD
hsa04136	**Autophagy - other**	3	31	2.05	1.56	**0.00035**	ATG7, MTOR, BECN1
hsa04140	**Autophagy - animal**	4	131	1.55	1.33	**0.00039**	ATG7, MTOR, BECN1, IRE1α/ ERN1
hsa05131	**Shigellosis**	4	218	1.33	0.98	**0.0022**	NLRP3, MTOR, BECN1, NLRC4
hsa05014	**Amyotrophic lateral sclerosis**	4	350	1.12	0.69	**0.0113**	RAB5A, MTOR, BECN1, IRE1α/ERN1
hsa05017	**Spinocerebellar ataxia**	3	135	1.41	0.79	**0.0113**	MTOR, BECN1, IRE1α/ERN1
**SCZ**							
hsa04621	**NOD-like receptorsignaling pathway**	9	173	1.76	3.6	**6.06E-12**	NLRP6, NLRP3, AIM2, CASP5, CASP4, GSDMD,CARD18, NLRP1, CARD8
hsa04140	**Autophagy - animal**	6	131	1.7	2.36	**2.94E-07**	RAB7A, RUBCN, ATG7, MTOR, BECN1, IRE1α/ERN1
hsa05132	**Salmonella infection**	6	209	1.5	1.84	**2.92E-06**	RAB7A, RAB5A, NLRP3, CASP4, GSDMD
hsa04136	**Autophagy - other**	3	31	2.02	1.55	**0.00032**	ATG7, MTOR, BECN1
hsa05131	**Shigellosis**	4	218	1.3	0.93	**0.0028**	NLRP3, MTOR, BECN1, CASP4
hsa05014	**Amyotrophic lateralsclerosis**	4	350	1.1	0.65	**0.0142**	RAB5A, MTOR, BECN1, IRE1α/ERN1
hsa05017	**Spinocerebellar ataxia**	3	135	1.39	0.74	**0.0142**	MTOR, BECN1, IRE1α/ERN1

This table shows significantly enriched KEGG pathways identified by STRING analysis, including the term identifier (ID), pathway description, observed gene count, background gene count, enrichment strength, signal, FDR-adjusted value, and matched proteins present in each network. Enrichment strength reflects the magnitude of enrichment relative to the expected background, whereas signal integrates enrichment magnitude and statistical significance. FDR-adjusted values indicate enrichment significance after correction for multiple testing. FDR: false discovery rate; STRING: Search Tool for the Retrieval of Interacting Genes/Proteins

## Discussion

Inflammatory mechanisms have been extensively associated with neuropsychiatric and stress-related diseases, including MDD and SCZ [15, 16]. Autophagy impairment has also been described [17, 18], along with prolonged ER stress and UPR irregularities that might contribute to both pathologies [19]. Here, we analyzed gene and protein expression alterations of selected inflammatory, autophagy-related, and UPR mediators in the DLPFC of MDD and SCZ subjects compared with matched controls. Our results reveal a dissociation between gene transcription and protein abundance in these pathways. IRE1α emerged as the most consistent shared alteration, showing increased transcription in both disorders but reduced protein abundance, particularly in SCZ. Sex-stratified analyses determined differential gene and protein patterns, pointing to MDD men and SCZ women as the most vulnerable groups. Age-related changes particularly occurred in autophagy and UPR pathways in SCZ, especially at mRNA level. Lastly, four genes and one protein showed distinct regulation in suicidal SCZ subjects.

In this study, transcription-protein dissociation refers to the lack of direct correspondence between mRNA abundance and the levels of the corresponding protein. This interpretation is supported by the absence of significant Spearman correlations between gene and protein layers. Such dissociation is progressively acknowledged in transcriptomic and proteomic studies, as protein abundance is shaped not only by mRNA levels but also by post-transcriptional regulation, translation efficiency, and protein turnover [20, 21].

Logistic regression analyses revealed that increased transcription of several inflammatory (*NLRP3*, *AIM2*), autophagy (*ATG7*, *RAB7*), and UPR-related (*IRE1α*) genes was associated with higher odds of MDD and SCZ, whereas lower protein levels of these markers were linked to reduced disease probability. This mRNA-protein dissociation may reflect multilayer buffering of cellular stress responses rather than proportional translation into protein output [22–24]. In MDD and SCZ, such regulation may involve post-transcriptional and/or post-translational regulatory mechanisms in the diseased brain [25, 26], including changes in mRNA stability, localization, translation efficiency, RNA-binding protein activity, microRNAs, and alternative splicing [27, 28]. This may be particularly relevant for autophagy-related genes, as *ATG7* mRNA stability can be regulated by the RNA-binding protein HuR [29]. Thus, coordinated transcriptional alterations may reflect a common cellular stress response, whereas reduced protein abundance may indicate downstream restrain of pathway activation [30], possibly contributing to cellular homeostasis under chronic neuroinflammatory and stress-related stimuli associated with MDD and SCZ [31, 32]. Chronic ER stress may also attenuate global protein synthesis through PERK-eIF2α signaling, whereas IRE1α can modulate RNA fate through regulated IRE1-dependent decay [33, 34]. Furthermore, ubiquitin–proteasome degradation, autophagy-lysosomal turnover, ER-associated degradation, proteolytic cleavage, or post-translational modifications may further reduce protein abundance despite increased transcription [35, 36]. Therefore, the mRNA-protein dissociation observed here may reflect multilayer regulation of proteostasis, inflammatory signaling, and cellular stress responses in the diseased brain.

The STRING-based analysis is consistent with functional crosstalk among the three molecular routes analyzed. In both pathologies, disease-associated markers converged on NOD-like receptor signaling and autophagy-related pathways, indicating that these factors form interconnected functional modules rather than independent alterations [37]. Notably, SCZ showed a distinct enrichment hierarchy, with autophagy-related terms ranking higher and including a broader vesicular trafficking/proteostasis module. This architecture is consistent with a more extensive disruption of autophagy-endosomal trafficking and proteostatic regulation in SCZ [10, 38], while both MDD and SCZ retain a shared inflammasome-UPR-autophagy functional backbone [39].

Among all markers, IRE1α was one of the most consistently altered, highlighting this UPR stress sensor as a potentially relevant integrative node in MDD and SCZ. The ANCOVA-based analysis showed that the relationship between IRE1α transcription and protein abundance was not explained by a direct within-group linear correlation. This disease-dependent shift in protein abundance was more pronounced in SCZ, consistent with the broader molecular disruption detected in this disorder [40] and with possible downstream regulation affecting translation, protein stability, or ER stress adaptation [14]. This is particularly relevant because IRE1α acts at the interface between transcriptional and post-transcriptional regulation: beyond its canonical role in ER stress signaling, IRE1α connects XBP1-mediated UPR activation with selective remodeling of mRNA availability and downstream protein output [34, 41, 42], and contributes to inflammatory signaling through NLRP3 assembly and activation [43, 44]. Therefore, IRE1α lies at the intersection of the three pathways studied and may help explain the transcription-protein dissociation observed across inflammatory, autophagy-related, and UPR markers. Curiously, reduced IRE1α has been proposed as a potential antidepressant mechanism of S-ketamine in rats [45], while increased mRNA levels of UPR markers have been reported in the DLPFC of MDD subjects and linked to suicidal behavior [46]. Further studies are warranted to clarify the central regulatory role of IRE1α in MDD and SCZ and its potential use as disease biomarker [14, 47].

ATG7 may also illustrate multilayer regulation. Increased transcription may reflect an attempt to activate autophagy under disease-related stress, whereas reduced protein abundance could indicate impaired translation, enhanced turnover, or insufficient stabilization of the autophagy machinery [48]. This interpretation is supported by evidence showing that ATG7 is regulated by non-coding RNAs, RNA-binding proteins, and post-translational modifications [49].

Specifically, *NLRP3* upregulation was correlated with higher risk for MDD, while *AIM2* was linked to SCZ. As these inflammasomes activate distinct innate immune pathways [50], our results suggest differential inflammasome involvement across disorders. In contrast to the transcription-protein dissociation observed for most markers, RAB5 showed a concordant decrease at both levels, associated with reduced odds of SCZ. Given the central role of RAB5 in early endosome trafficking and receptor sorting [51–53], this finding may suggest that attenuation of RAB5-dependent endosomal activity is linked to a less disease-prone molecular state in SCZ, although causality cannot be established from these data.

This study also highlights sex-dependent molecular patterns in MDD and SCZ. Our findings align with previous evidence of sexual dimorphism in psychiatric disorders [54, 55], likely involving differences in immune regulation, hormonal signaling, and stress-response pathways [56–58]. Consistently, inflammatory and autophagy-related proteins showed stronger associations in MDD men, suggesting sex-specific contributions of these pathways to disease vulnerability [59–61]. The broader transcriptional alterations observed in SCZ women also agree with reports of greater transcriptomic dysregulation in female brains across several psychiatric disorders, including SCZ [62], and with evidence that sex-specific transcriptomic signatures may involve distinct brain cell types in the depressive brain [63].

Regarding age, our findings suggest that the physiological age-dependent regulation of cellular stress responses (*NRF2*, *CHOP*) and autophagy activity (*BECLIN1*, *LAMP2A*, *SQSTM1*, *ULK1*) may be altered in MDD and SCZ. In healthy individuals, transcriptional modulation reflects adaptive mechanisms maintaining neuronal homeostasis during aging [64–66]. In contrast, these patterns appeared attenuated or reshaped in MDD and particularly in SCZ, where additional age-associated changes emerged in *SQSTM1* and *ULK1*. In contrast, protein-level age effects were limited and mainly observed in inflammatory mediators in SCZ, suggesting that aging may interact with neuroinflammatory pathways in this disorder. Together, these observations support that psychiatric conditions may disrupt normal age-related regulation of cellular stress and immune pathways in the brain.

Altered *IRE1α, CASPASE-5* and *IL18* transcription, along with CASPASE-8 protein, point towards enhanced inflammasome-related and apoptotic signaling in suicidal individuals. Suicidality has been linked to enhanced neuroinflammation in MDD patients [67, 68], which may indicate a stress-sensitized brain state that heightens vulnerability to suicide ideation and depressive affect [69]. These findings support a potential contribution of inflammatory and UPR pathways to suicide-related biology in SCZ. In addition, reduced *SQSTM1* transcription in suicidal SCZ subjects may indicate altered autophagy-related cellular homeostasis, a process functionally connected to neuroplasticity, cellular stress, and inflammatory responses previously associated with suicidality [70].

Publicly available transcriptomic, proteomic, and multiomic datasets provide independent support for our findings. The GSE53987 dataset reported broader differential gene expression in SCZ than in MDD across the prefrontal cortex, hippocampus, and striatum, including alterations in autophagy and cellular stress-related genes, including *ATG7*, *BECLIN1*, *NRF2*, and *CHOP* [71]. The GSE102556 dataset revealed marked sex-specific transcriptional signatures in MDD [54], while CommonMind Consortium and PsychENCODE resources further confirm widespread transcriptomic and regulatory dysregulation in the DLPFC of psychiatric disorders, particularly in SCZ [40, 72]. At the protein level, brain proteome-wide association studies show that psychiatric risk may be mediated through changes in brain protein abundance and that mRNA levels are not always reliable proxies for protein expression [21]. Together, these external resources support our main framework: stronger molecular disruption in SCZ, involvement of inflammatory and cellular stress pathways, and the need to integrate gene and protein analyses in postmortem human brain studies.

This work has several limitations. First, analyses were performed exclusively in postmortem brain tissue, which makes the study observational and prevents causal inference. Only a subset of markers was examined at the protein level due to limited tissue availability, and therefore additional protein alterations may have remained undetected. Another limitation concerns the relatively modest sample size. Although cases and controls were carefully matched for key variables to reduce potential confounding effects, the cohort remains smaller than those typically included in large population-based psychiatric studies. Sex-stratified analyses were also limited by unequal group distribution, as the female cohort was smaller than the male group, potentially reducing the statistical power. Another limitation is that all MDD subjects died by suicide, which may confound disease-related interpretation because some alterations may partly reflect suicide-related or perimortem factors. Future studies including non-suicide MDD cases are needed to distinguish disease-specific from suicide-related molecular alterations. Finally, residual effects of medication exposure and agonal state cannot be fully excluded, although toxicological screening was performed; PMI was included as a covariate in the statistical analyses and showed no significant effects.

In conclusion, this study provides evidence that key components of inflammatory, autophagy-related, and ER stress pathways are altered in the DLPFC of individuals with MDD and SCZ. Combined gene and protein analysis, together with stratification by sex, age, and cause of death, revealed complex regulatory patterns affecting these pathways. Although further studies are required to clarify their functional implications, these findings support a role for cellular stress and immune-related mechanisms in the neurobiology of MDD and SCZ.

## Materials and methods

### Postmortem human brain samples

Human brain samples were obtained at autopsy in the Basque Institute of Legal Medicine (Bilbao, Spain). DLPFC tissue (Brodmann’s area 9) was dissected at autopsy and immediately stored at −80ºC until assayed. Blood toxicological screening at the time of death, including antidepressants, antipsychotics, other psychotropic drugs, and ethanol, was performed at the National Institute of Toxicology (Madrid, Spain). All positive drug findings were confirmed by ultra-performance liquid chromatography coupled to tandem mass spectrometry (UPLC-MS/MS).

The cohort included 28 subjects with an antemortem diagnosis of MDD, 28 subjects with an antemortem diagnosis of SCZ (both based on DSM and ICD criteria), and 28 control subjects without known neurological or psychiatric disorders. Case and controls were carefully matched by age, sex, storage time, and postmortem interval (PMI; time interval between death and autopsy) to reduce potential confounding effects on gene and protein expression analyses. MDD and SCZ subjects were classified as antidepressant-free/treated or antipsychotic-free/treated according to the absence or presence of these drugs in blood at the time of death. As expected in postmortem psychiatric cohorts, medication exposure was heterogeneous and reflected clinical variability. Given this heterogeneity and the limited sample size, medication status was not included as a covariate; however, no clear medication-class pattern was observed that could account for the molecular alterations described. Demographic variables, PMI, and blood pharmacological screening data are provided in Table S6.

### RNA isolation and quantitative real-time polymerase chain reaction (RT-qPCR)

All samples were processed simultaneously to minimize technical variability. Total cytoplasmic RNA was extracted from DLPFC using TRIzol (Invitrogen, 15,596,026). RNA integrity was assessed by a 3’:5’ ratio assay via RT-qPCR using the *GAPDH* gene, confirming an appropriate RNA quality (ΔCt 0.2–0.8) [73]. Gene expression levels were quantified by real-time multiplex PCR using TaqMan probes, enabling the simultaneous amplification of four targets per reaction (5-FAM, HEX, Texas Red, and Cy5; Table S7). Reactions were performed in 10 µl using SsoAdvanced Universal Probes Supermix (BioRad, 1,725,280) and the CFX384 Touch Real-Time PCR Detection System (BioRad).

All samples were analyzed in triplicate with negative controls. Eight housekeeping genes were initially tested for stability; *β-ACTIN* and *UBE2D2* were the most consistent, and *UBE2D2* was used for normalization. Relative gene expression was calculated using the 2^−ΔΔCT^ method. Further methodological details are provided in the Supplementary Information.

### Protein extraction, immunoblotting, and ELISA

Pure cytosolic fractions from DLPFC were obtained following a previously described protocol [74]. For Western blot analyses, 12 µg of protein per sample were separated by SDS-PAGE and transferred to 0.2 µm nitrocellulose membranes (Bio-Rad, 1,704,159). Membranes were blocked and incubated overnight at 4ºC with primary antibodies (Table S8), followed by HRP-conjugated secondary antibodies. Proteins were revealed using an ECL® kit and visualized using the ChemiDoc Imaging System (BioRad). Protein levels were quantified by densitometry using ImageJ, with β-ACTIN as loading control.

Additional protein determinations were performed by ELISA after tissue sonication in PBS containing protease and phosphatase inhibitors. Commercial kits were used to measure CASPASE-1, CASPASE-5, CASPASE-8, IL1β, IL18, and PROTEASOME 26S. Further methodological details are provided in the Supplementary Information.

### Data analysis

The potential association between gene or protein expression and diagnosis, MDD or SCZ, were analyzed using a binary logistic regression (Table 1). Data distribution is shown in Table S9. Potential sex and age effects were examined through stratified analyses. The cohort median age was 35 years and was therefore used as the cutoff for age-stratified gene and protein analyses (Tables 2 and 3).

When complete or quasi-complete separation caused model non-convergence or unstable standard errors, Firth’s penalized likelihood logistic regression was employed. This method provides reliable estimates of odds ratios (*OR*), 95% confidence intervals (CI), and *p* values in small sample sizes and separated data. Linear regression models were applied to evaluate age-related associations with gene or protein expression in controls, MDD and SCZ subjects.

To further evaluate the nature of the changes, two-tailed Student’s *t*-tests were performed between controls and MDD or SCZ, including sex- and age-stratified comparisons. When a normality could not be assumed, the Mann–Whitney test was applied. Two-sided *p* values < 0.05 were considered statistically significant.

Subsequently, Spearman’s rank correlation was used to evaluate the relationship between mRNA and protein levels for markers quantified at both molecular layers (Table S5). Group differences in protein levels were also analyzed by ANCOVA, including disease status as the main factor, gene expression as a covariate, and their interaction term. Only IRE1α analysis is shown (Fig. 4a).

To assess the functional relationship among the disease-associated markers identified by logistic regression analyses, protein–protein interaction networks were generated using the STRING database [75]. MDD and SCZ input lists were analyzed independently, and network expansion was restricted to a maximum of 10 first-shell interactors to maintain a focused pathway-level analysis (Table 4).

Lastly, False Discovery Rate (FDR) correction via Benjamini–Hochberg method [76] to all *p* values derived from the models (both mRNA and protein datasets) are shown as *q* values in Table S10.

Because ELISA-derived protein values had different magnitude scales, they were multiplied by 1000 before analysis. Logistic and linear regression models were calculated using R, while the remaining univariate analyses and normality tests were performed with GraphPad Prism 10 (GraphPad Software, USA).

## Supplementary Information

Supplementary Material 1

## Data Availability

The data supporting the findings of this study are not openly available due to the sensitive nature of individual-level human postmortem brain data and associated clinical and postmortem metadata. De-identified data may be made available from the corresponding authors upon request, subject to applicable ethical, legal, and institutional requirements. Data are stored in controlled-access institutional repositories at the Universidad Complutense de Madrid.
