# Supplementary material for: Transcription-protein dissociation reveals disrupted cellular stress pathways in the prefrontal cortex of depression and schizophrenia subjects
Source: Mol Biomed. 2026 Aug 17;7:138. doi: 10.1186/s43556-026-00514-4 (PMC13481630; doi:10.1186/s43556-026-00514-4)
Supplement: Supplementary file 1 [file 43556_2026_514_MOESM1_ESM.docx]

**Transcription-protein dissociation reveals disrupted cellular stress pathways in the prefrontal cortex of depression and schizophrenia subjects**

Cristina Ulecia-Morón^1,2^*, Álvaro G. Bris^1,2^, Lucía Inglada-Pérez^3^, Luis F. Callado^2,4,5^, J. Javier Meana^2,4,5^, David Martín-Hernández^1,2^, Karina S. MacDowell^1,2^, Elena Figuero^6^, Javier R. Caso^1,2^*, Juan C. Leza^1,2^

^1^ Department of Pharmacology and Toxicology, Faculty of Medicine, University Complutense Madrid (UCM); Research Institute of Hospital 12 de Octubre (Imas12); University Institute of Research in Neurochemistry of the UCM (IUIN-UCM). Madrid, Spain

^2^ Biomedical Research Network Centre in Mental Health, Institute of Health Carlos III (CIBERSAM, ISCIII). Madrid, Spain.

^3^ Department of Statistics and Operational Research, Faculty of Medicine, UCM. Madrid, Spain.

^4^ Department of Pharmacology, University of the Basque Country, UPV/EHU, Leioa, Bizkaia, Spain.

^5^ BioBizkaia Health Research Institute, Barakaldo, Bizkaia, Spain.

^6^ ETEP (Etiology and Therapy of Periodontal and Peri-implant Diseases) Research Group; Department of Dental Clinical Specialties, Faculty of Dentistry, UCM, Madrid, Spain.

**Supplementary Materials**

**RNA isolation and quantitative real-time polymerase chain reaction (RT-qPCR)**

All samples were processed simultaneously to minimize technical variability. Total cytoplasmic RNA was obtained from DLPFC using TRIzol (Invitrogen, 15596026). To assess RNA integrity across all samples, a 3’:5’ ratio assay was performed via RT-qPCR using the *GAPDH* housekeeping gene as a reference. In all analyzed samples, the observed ΔCt values were consistently low, ranging between 0.2 and 0.8 cycles. This quality control measure ensured that the samples analyzed were suitable for reliable molecular analyses. Aliquots of each sample were then converted to cDNA by means of random hexamer primers. mRNA levels were quantitatively measured by real-time multiplex polymerase chain reaction assays. The multiplex technology allowed the amplification of several DNA sequences concurrently using TaqMan fluorescent probes. Four genes were amplified and analyzed in parallel from the same sample, thanks to the combination of four different fluorescent probes: 5-FAM (λ_ex/em_= 492/517 nm), HEX (λ_ex/em_= 530/556 nm), Texas Red (λ_ex/em_= 596/615 nm), and Cy5 (λ_ex/em_= 650/670 nm).

Primers were designed and manufactured by BioRad (PrimePCR^TM^ Probe Assay, one for each gene) (Table S7). Multiplex reactions were carried out in the presence of SsoAdvanced Universal Probes Supermix (BioRad, 1725280) with a total sample volume of 10 µl in the CFX384 Touch Real-Time PCR Detection System (BioRad). The cycling conditions were an initial denaturation at 95 ºC for 2 min and 45 cycles of 5 s at 95 ºC followed by 30 s at 60 ºC.

Triplicate measurements of each sample were assayed, together with negative internal controls in each run. Eight housekeeping genes were first tested to verify their homogeneity and consistency within all samples (*α-TUBULIN*, *β-ACTIN*, *CYC1*, *EIF4A2*, *GAPDH*, *TBP*, *PGK1*, and *UBE2D2*). Given the consistency and stability of *β-ACTIN* and *UBE2D2*, both were selected to normalize data. As no differences were found in the expression of *β-ACTIN* and *UBE2D2* levels among the examined groups, data normalization by *UBE2D2* is presented in this paper. mRNA concentration was calculated using the 2^-ΔΔCT^ method. Absolute levels of each gene are shown to further compare average expression levels.

**Protein extraction, immunoblotting, and ELISA**

Pure cytosolic fractions were obtained without nuclear interference by following a widely used protocol. Briefly, DLPFC samples were homogenized using an in-house buffer (10 mmol/l N-2-hydroxyethylpiperazine-N-2-ethanesulfonic acid [pH 7.9], 1 mmol/l EDTA, 1 mmol/l EGTA, 10 mmol/l KCl, 1 mmol/l dithiothreitol, 0.5 mmol/l phenylmethylsulfonyl fluoride, 0.1 mg/ml aprotinin, 1 mg/ml leupeptin, 1 mg/ml Na-p-tosyll-lysine-chloromethylketone, 5 mmol/l NaF, 1 mmol/l NaVO_4_, 0.5 mol/l sucrose, and 10-mmol/l Na_2_MoO_4_). Nonidet P-40^®^ (Sigma-Aldrich, 74385) was added to achieve a 0.5% concentration after 15 min. Samples were then vigorously vortexed for 15 s twice, and nuclei were centrifuged at 8000 *x g* for 5 min. Supernatants were quantified using the Bradford method and used as cytosolic fractions in Western blot analyses. Once protein levels were adjusted and mixed with *Laemmli* sample buffer (Bio-Rad, 1610737) and β-mercaptoethanol, 12 µg of protein from each sample were loaded into SDS-polyacrylamide gels (CliniSciences, DG102-008-15w-02) to carry out electrophoresis (100 V). Gels were transferred onto 0.2 µm nitrocellulose membranes (Bio-Rad, 1704159) using a semi-dry transfer system (Bio-Rad, USA). Membranes were blocked in Tris-buffered saline 1x (TBS 10x: 200 mM Tris, 1500 mM NaCl, pH 7.6) supplemented with 0.1% Tween 20 (BioRad, 1706531) and 5% Bovine Serum Albumin (BSA; PanReac AppliChem, A6588). Primary antibodies were incubated overnight at 4 ºC (Table S8). The next day, once membranes were washed three times with TBS containing 0.1% Tween 20, HRP-conjugated secondary antibodies were incubated for 1h 30 min at room temperature. Then, three more washes were performed, and membranes were revealed with ECL® kit following manufacturer’s instructions (Fisher Scientific, 12994780) using the ChemiDoc Imaging System (BioRad). The loading housekeeping control was β-ACTIN. Each assayed protein was repeated at least three times in separate assays, to ensure the reproducibility and linearity of the results. Protein bands were quantified by densitometry using the NIH ImageJ® software. Results are expressed as arbitrary units of optical density as a percentage normalized to the control group.

More complex protein determinations were carried out by ELISA. In this case, homogenization of DLPFC was achieved by sonication in PBS mixed with a cocktail of protease and phosphatase inhibitors (Roche, 11836170001 and 4906845001). Protein concentration was then determined by the Bradford method and adjusted to the appropriate dilutions. Commercially available ELISA kits were employed to measure CASPASE 1 (Elabscience, E-EL-H0016), CASPASE 5 (BioTechne, NBP3-31200), CASPASE 8 (Cusabio, CSB-E13636h), IL1β (CloudClone®, SEA563Hu), IL18 (CloudClone®, SEA064Hu), and PROTEASOME 26S protein levels (FineTest, EH2076), following the manufacturer’s instructions.

**Supplementary Results**

**Fig. S1**

**Sex differences in autophagy, inflammatory, and UPR genes in MDD (a-e) and SCZ (f-k).** All genes were individually analyzed and compared according to sex using the Student’s *t*-test (Table S2); only those with significant alterations are shown. Men are depicted in orange and women in purple. Violin plots illustrate the kernel density estimation of the data distribution; the width of the shaded area is proportional to the frequency of observations. The horizontal dashed lines represent the mean, while the dotted lines indicate the first and third quartiles (interquartile range). Violin plots were used to better visualize data distribution due to the relatively large number of observations in the group of men (men, n=19; women, n=9). A *p<0.05 was considered statistically significant (***p*<0.01, ****p*<0.001).

**Fig. S2**

**Sex differences in inflammation (a-g), autophagy (h-o), and UPR (p-r) proteins in MDD and SCZ.** Selected proteins were individually analyzed and compared according to sex using the Student’ *t*-test (Table S2); only those with significant alterations are shown. Men are depicted in orange and women in purple. Violin plots illustrate the kernel density estimation of the data distribution; the width of the shaded area is proportional to the frequency of observations. The horizontal dashed lines represent the mean, while the dotted lines indicate the first and third quartiles (interquartile range). A **p*<0.05 was considered statistically significant (***p*<0.01, ****p*<0.001, *****p*<0.0001).

**Fig. S3**

**Age-associated tendencies in inflammatory, autophagy-related, and UPR marker gene expression.** Selected markers showing marginal age-derived changes in controls, MDD, or SCZ subjects are represented. Gene expression was analyzed using linear regression, and tendencies close to statistical significance are indicated in each graph. *ATF4* and *BID* tended to decrease with age in controls (a-f), whereas *CASPASE-1* showed a similar tendency only in SCZ subjects (g-i). *IF2α* tended to increase with age in MDD individuals (j-l), while *mTOR* showed a similar pattern in SCZ subjects (m-o). *PERK* tended to increase with age in controls (p-r), whereas *RAB5* tended to decrease with age in MDD subjects, but not in controls (s-u). White dots represent controls (first column), blue dots illustrate MDD subjects (second column), and red dots exhibit SCZ patients (third column).

**Fig. S4**

**Age-related differences in autophagy, inflammatory, and UPR gene expression in MDD and SCZ.** Genes were compared between age groups using Student’s *t*-test (Table S3), and only significant alterations are shown. Subjects were stratified using 35 years as the cutoff, corresponding to the cohort median. Graphs show significant changes in MDD (a-c) and SCZ (d-i). Olive green represents subjects <35 years, and teal depict subjects ≥35 years. Violin plots illustrate the data distribution, with dashed lines representing the mean and the dotted lines indicating the first and third quartiles (n=14 in each group). A **p*<0.05 was considered statistically significant (***p*<0.01, ****p*<0.001).

**Fig. S5**

**Age-associated differences in inflammatory, autophagy-related, and UPR proteins in MDD and SCZ.** Proteins were compared between age groups using Student’s *t*-test (Table S3), and only significant alterations are shown. Subjects were stratified using 35 years as the cutoff, corresponding to the cohort median. Graphs show inflammatory (a-g), autophagy-related (h-n), and UPR protein (o-p) alterations. Olive green represents subjects <35 years, and teal depict subjects ≥35 years. Violin plots illustrate the data distribution, with dashed lines representing the mean and the dotted lines indicating the first and third quartiles (n=14 in each group). A **p*<0.05 was considered statistically significant (***p*<0.01, ****p*<0.001).

**Table S1. Statistical analysis of protein expression differences in MDD and SCZ subjects.**

| PROTEINS | MDD | SCZ |
| --- | --- | --- |
| INFLAMMATION | | |
| AIM2 | F=1.117; t=3.868; df=53; **p=0.0003** | F=1.912; t=3.479; df=54; **p=0.001** |
| CASPASE 1 | F=1.378; t=1.491; df=52; p=0.1419 | F=2.519; t=1.448; df=45,95; p=0.1545 |
| CASPASE 5 | U=272; ranks: 862,623; p=0.1137 (exact) | U=178; ranks: 984,556; **p=0.006** (exact) |
| CASPASE 8 | F=1.441; t=0.08398; df=54; p=0.9334 | F=1.301; t=0,3290; df=54; p=0.7435 |
| IL1β | F=1.385; t=0,7146, df=52; p=0.4781 | F=1.982; t=1,792, df=53; p=0.0789 |
| IL18 | U=348; ranks: 759,726; p=0.7437 (exact) | U=256; ranks: 634,906; **p=0,0401** (exact) |
| NLRC4 | F=1.651; t=2,520; df=53; **p=0.0148** | U=243; ranks: 864,621; **p=0.0356** (exact) |
| NLRP3 | U=185; ranks:1005,591; **p=0.0005** (exact) | U=90; ranks:1100,496; **p<0.0001** (exact) |
| AUTOPHAGY | | |
| ATG7 | U=159; ranks: 1031,565; **p<0,0001** (exact) | U=94; ranks: 1096,500; **p<0.0001** (exact) |
| mTOR | U=165; ranks: 1025,571; **p=0.0001** (exact) | U=156; ranks: 1034,562; **p<0.0001** (exact) |
| RAB5A | F=1.829; t=4,663; df=54; **p<0.0001** | F=1.730; t=5,370; df=54; **p<0.0001** |
| RAB7 | U=297; ranks:893,703; p=1.221 (exact) | U=226; ranks:964,632; **p=0.0060** (exact) |
| UPR | | |
| IRE1α | F=1.853; t=7,178; df=53; **p<0.0001** | F=2.001; t=13,92, df=53; **p<0.0001** |
| PROTEASOME 26S | F=1.438; t=1,563, df=52; p=0.1240 | U=278; ranks: 656,884; p=0.0941 (exact) |

This table presents the analysis performed using Student’s *t*-test or the Mann-Whitney test across the studied proteins.

**Table S2. Statistical analysis of sex differences between men and women with MDD and SCZ at mRNA and protein levels.**

|  | Men | | Women | |
| --- | --- | --- | --- | --- |
| Genes | **MDD** | **SCZ** | **MDD** | **SCZ** |
|  | **INFLAMMATION** | | | |
| *AIM2* | F=3.412; t=1.812; df=25.87; p=0.0816 | U=125; ranks:315,426; p=0.1090 (exact) | U=39; ranks:87,84; p=0.931 (exact) | U=28; ranks:73,98; p=0.2973 (exact) |
| *ASC* | U=168; ranks:358,383; p=0.7291 (exact) | U=153; ranks:343,398; p=0.435 (exact) | F=1.046; t=1.014; df=16; p=0.3255 | U=18; ranks:108,63; p=0.0503 (exact) |
| *BID* | U=156; ranks:346,395; p=0.4879 (exact) | U=156; ranks:346,357; p=0.663 (exact) | F=1.629; t=0.938; df=16; p=0.362 | F=3.247; t=0.968; df=15; p=0.349 |
| *CASPASE 1* | U=154; ranks:397, 344; p=0.4523 (exact) | F=1.583; t=0.4735; df=36; p=0.6387 | U=28; ranks:80, 73; p=0.4807 (exact) | U=34; ranks:74,79; p=0.8884 (exact) |
| *CASPASE 2* | U=155; ranks:345,396; p=0.47 (exact) | U=165; ranks:355,348; p=0.869 (exact) | F=1.776; t=2.01; df=16; p=0.062 | F=7.297; t=1.546; df=8.696; p=0.158 |
| *CASPASE 4* | U=162; ranks:389,352; p=0.6032 (exact) | F=4.279; t=1.406; df=25.98; p=0.1715 | U=36; ranks:81,90; p=0.7304 (exact) | U=31; ranks:76,95; p=0.4363 (exact) |
| *CASPASE 5* | U=151; ranks:341,400; p=0.4016 (exact) | U=119; ranks:309,432; p=0.0747 (exact) | F=13.33; t=0.9332; df=18; p=0.3631 | F=11.4; t=0.743; df=10.47; p=0.4737 |
| *CASPASE 8* | U=169; ranks:382,359; p=0.751 (exact) | U=179; ranks:369,372; p=0.977 (exact) | U=15; ranks:60,111; **p=0.0244** (exact) | U=36; ranks:81,72; p>0.9999 (exact) |
| *IL1β* | U=174; ranks:364,377; p=0.8626 (exact) | U=151; ranks:341,400; p=0.4016 (exact) | F=743.4; t=1.19; df=8.022; p=0.2682 | F=2.353; t=1.745; df=16; p=0.1002 |
| *IL18* | U=171; ranks:380,361; p=0.7951 (exact) | U=158; ranks:348,393; p=0.525 (exact) | F=3.313; t=0.7617; df=16; p=0.4573 | U=22; ranks:104,67; p=0.1135 (exact) |
| *NALP1* | U=167; ranks:365,338; p=0.9163 (exact) | U=126; ranks:316,425; p=0.116 (exact) | F=1.454; t=0.252; df=16; p=0.804 | F=2.956; t=0.536; df=16; p=0.5995 |
| *NLRC4* | U=143; ranks:333,370; p=0.408 (exact) | F=2.545; t=0.073; df=36; p=0.942 | U=25; ranks:61,92; p=0.3213 (exact) | U=28; ranks:64,89; p=0.481 (exact) |
| *NLRP3* | U=108; ranks:298,405; p=0.0569 (exact) | F=2.704; t=1.281; df=20; p=0.2147 | F=10.25; t=0.673; df=9.546; p=0.5171 | F=16.73; t=0.439; df=8.953; p=0.671 |
| *NRF2* | U=175; ranks:365,376; p=0.8853 (exact) | U=142; ranks:332,409; p=0.271 (exact) | F=2.102; t=0.5556; df=16; p=0.5862 | F=3.481; t=1.704; df=16; p=0.1078 |
| *TXNIP* | U=179; ranks:372,369; p=0.977 (exact) | U=137; ranks:327,376; p=0.313 (exact) | U=29; ranks:74,97; p=0.34 (exact) | F=1.627; t=0.884; df=15; p=0.391 |
|  | **AUTOPHAGY** | | | |
| *ATG3* | U=153; ranks:398,343; p=0.435 (exact) | F=1.242; t=0.362; df=36; p=0.719 | F=2.453; t=0.627; df=16; p=0.539 | F=2.002; t=0.047; df=16; p=0.963 |
| *ATG7* | F=2.082; t=3.639; df=36; **p=0.0009** | F=2.954; t=0.8443; df=28.93; p=0.4054 | F=2.432; t=2.034; df=16; p=0.059 | F=2.222; t=1.548; df=16; p=0.1411 |
| *ATG16L1* | U=104; ranks:294,447; **p=0.0253** (exact) | F=1.873; t=0.5882; df=36; p=0.5601 | F=1.706; t=0.759; df=15; p=0.459 | F=3.66; t=0.0126; df=15; p=0.9901 |
| *BECLIN1* | F=1.635; t=1.485; df=36; p=0.1461 | U=157; ranks:347,394; p=0.5063 (exact) | F=1.293; t=0.6182; df=16; p=0.5452 | F=1.376; t=1.833; df=16; p=0.0854 |
| *CYSTATIN C* | U=140; ranks:411,330; p=0.246 (exact) | U=134; ranks:324,417; p=0.181 (exact) | F=1.808; t=0.057; df=16; p=0.956 | F=2.323; t=1.593; df=16; p=0.131 |
| *GALECTIN 3* | U=170; ranks:360,381; p=0.773 (exact) | U=125; ranks:315,426; p=0.1090 (exact) | F=4.493; t=0.173; df=11.39; p=0.8658 | U=28; ranks:73,98; p=0.2973 (exact) |
| *GALECTIN 9* | F=1.015; t=1.619; df=36; p=0.1142 | F=2.677; t=0.4191; df=29.8; p=0.6781 | F=1.264; t=0.2079; df=16; p=0.838 | F=1.083; t=0.5915; df=16; p=0.5624 |
| *LAMP2A* | U=161; ranks:390,351; p=0.583 (exact) | U=147; ranks:404,337; p=0.339 (exact) | U=33; ranks:78,93; p=0.546 (exact) | U=32; ranks:94,77; p=0.489 (exact) |
| *LC3α* | F=3.128; t=1.867; df=28.44; p=0.072 | F=2.336; t=1.007; df=36; p=0.3204 | F=1.188; t=0.531; df=15; p=0.603 | F=1.529; t=1.937; df=15; p=0.0718 |
| *mTOR* | F=2.637; t=1.289; df=29.93; p=0.2072 | F=1.287; t=1.073; df=36; p=0.2903 | F=1.33; t=0.31; df=16; p=0.7606 | F=1.311; t=0.4719; df=16; p=0.6434 |
| *RAB5* | F=3.468; t=0.712; df=27.58; p=0.4823 | U=164; ranks:387,354; p=0.644 (exact) | F=1.494; t=1.183; df=16; p=0.254 | F=1.301; t=2.712; df=16; **p=0.0154** |
| *RAB7* | F=3.747; t=3.404; df=26.97; **p=0.0021** | U=105; ranks:295,446; **p=0.0273** (exact) | F=2.904; t=0.852; df=16; p=0.407 | F=1.011; t=0.907; df=16; p=0.378 |
| *SQSTM1* | U=155.5; ranks:345.5,395.5; p=0.475 (exact) | U=154.5; ranks:344.5,396.5; p=0.4567 (exact) | F=1.211; t=0.836; df=16; p=0.415 | U=25; ranks:70,101; p=0.1903 (exact) |
| *ULK1* | F=1.464; t=0.929; df=36; p=0.3592 | U=169; ranks:382,359; p=0.7509 (exact) | U=32; ranks:77,94; p=0.4894 (exact) | U=39; ranks:84,87; p=0.9314 (exact) |
|  | **UPR** | | | |
| *ATF4* | F=1.404; t=0.4577; df=36; p=0.6499 | U=171; ranks:361,380; p=0.7951 (exact) | F=1.489; t=1.042; df=16; p=0.3127 | F=1.681; t=2.606; df=16; **p=0.0191** |
| *ATF6* | U=156; ranks:346,395; p=0.4879 (exact) | F=3.824; t=0.8542; df=26.81; p=0.4006 | U=38; ranks:83,88; p=0.8633 (exact) | U=30; ranks:96, 75; p=0.3865 (exact) |
| *BDNF* | U=;158 ranks:348;393 p=0.525 (exact) | F=2.455; t=0.838; df=36; p=0.408 | U=38; ranks:88,83; p=0.8633 (exact) | U=15; ranks:93,60; **p=0.0464** (exact) |
| *BiP* | F=2.634; t=0.7818; df=29.95; p=0.4405 | U=166; ranks:385,356; p=0.6861 (exact) | F=3.129; t=0.0235; df=16; p=0.9816 | F=2.424; t=1.896; df=16; p=0.0761 |
| *CHOP* | U=121; ranks:430,311; p=0.085 (exact) | U=159; ranks:392,349; p=0.5441 (exact) | F=1.572; t=0.6643; df=16; p=0.5159 | F=4.164; t=0.0083; df=16; p=0.9935 |
| *IF2α* | U=180; ranks:370,371; p>0.9999 (exact) | U=166; ranks:356,385; p=0.6861 (exact) | U=33; ranks:93,78; p=0.5457 (exact) | U=29; ranks:74,97; p=0.3401 (exact) |
| *IRE1α* | F=1.001; t=4.194; df=36; **p=0.0002** | F=2.242; t=3.999; df=36; **p=0.0003** | F=1.636; t=2.271; df=16; **p=0.0373** | F=4.013; t=2.69; df=16; **p=0.0161** |
| *PERK* | U=124; ranks:314,427; p=0.1025 (exact) | U=155; ranks:345,396; p=0.4699 (exact) | F=1.536; t=0.0164; df=16; p=0.9871 | F=2.369; t=1.884; df=16; p=0.0779 |
| *XBP1* | U=166; ranks:356,385; p=0.6861 (exact) | U=146; ranks:336,405; p=0.3249 (exact) | U=39; ranks:87,84; p=0.9314 (exact) | F=2.097; t=3.222; df=16; **p=0.0053** |
|  | **PROTEINS** | | | |
|  | **INFLAMMATION** | | | |
| AIM2 | F=1.29; t=3.57; df=35; **p=0.0011** | F=1.942; t=3.097; df=36; **p=0.0038** | F=1.311; t=1.521; df=16; p=0.1479 | F=1.797; t=1.523; df=16; p=0.1472 |
| CASPASE 1 | F=1.414; t=2.364; df=34; **p=0.0239** | F=2.221; t=1.136; df=34; p=0.2641 | F=1.024; t=0.7213; df=16; p=0.4811 | F=3.106; t=0.8915; df=16; p=0.3858 |
| CASPASE 5 | F=1.095; t=1.491; df=36; p=0.1447 | F=2.665; t=3.484; df=30.09; **p=0.0015** | F=3.333; t=0.6623; df=14; p=0.5185 | F=2.767; t=1.57; df=16; p=0.1361 |
| CASPASE 8 | F=1.129; t=0.6796; df=36; p=0.5011 | F=1.168; t=0.2445; df=36; p=0.8082 | F=1.459; t=1.217; df=16; p=0.2413 | F=2.018; t=0.2331; df=16; p=0.8186 |
| IL1β | F=1.046; t=0.65; df=34; p=0.5202 | F=2.012; t=1.623; df=35; p=0.114 | F=1.65; t=2.241; df=15; **p=0.0406** | F=2.093; t=0.7341; df=16; p=0.474 |
| IL18 | U=147; ranks:318,348; p=0.6503 (exact) | U=111; ranks:282,421; p=0.0703 (exact) | U=26; ranks:91,62; p=0.3704 (exact) | F=2.13; t=1.356; df=16; p=0.194 |
| NLRC4 | F=2.843; t=1.759; df=29.54; p=0.0889 | F=1.63; t=1.888; df=34; p=0.0676 | F=2.509; t=1.924; df=16; p=0.0724 | F=1.092; t=1.288; df=16; p=0.2159 |
| NLRP3 | U=81; ranks:470,271; **p=0.0031** (exact) | U=45; ranks:506,235; **p<0.0001** (exact) | F=1.708; t=2.239; df=16; **p=0.0397** | F=1.313; t=3.651; df=16; **p=0.0022** |
| AUTOPHAGY | | | | |
| ATG7 | U=58; ranks:493,248; **p=0.0002** (exact) | U=31; ranks:520,221; **p<0.0001** (exact) | U=22; ranks:104,67; p=0.1135 (exact) | U=16; ranks:110,61; **p=0.0315** (exact) |
| mTOR | U=70; ranks:481,260;  **p=0,0009** (exact) | U=72; ranks:479,262; **p=0.0011** (exact) | F=1.912; t=1.817; df=16; p=0.0879 | F=3.638; t=2.418; df=16; **p=0.0279** |
| RAB5A | F=2.253; t=4.034; df=36; **p=0.0003** | F=2.247; t=4.908; df=36; **p<0.0001** | F=1.596; t=2.454; df=16; **p=0.0259** | F=1.106; t=2.358; df=16; **p=0.0314** |
| RAB7 | F=1.277; t=2.968; df=36; **p=0.0053** | F=1.35; t=3.092; df=36; **p=0.0038** | F=6.766; t=0.3297; df=11.6; p=0.7475 | F=7.656; t=0.2677; df=10.05; p=0.7944 |
| UPR | | | | |
| IRE1α | U=25; ranks:488,215; **p<0.0001** (exact) | F=1.537; t=12.92; df=35; **p<0.0001** | F=1.604; t=2.774; df=16; **p=0.0135** | F=2.545; t=6.895; df=16; **p<0.0001** |
| PROTEASOME 26S | F=1.294; t=0.0139; df=34; p=0.989 | F=1.533; t=1.435; df=35; p=0.1603 | F=2.509; t=2.942; df=15; **p=0.0101** | F=1.672; t=1.116; df=16; p=0.281 |

This table presents the analysis performed using Student’s *t*-test or the Mann-Whitney test when stratifying by sex gene and protein-level results.

**Table S3. Statistical analysis of age-associated differences in MDD and SCZ subjects at the mRNA and protein levels.**

|  | < 35 years old | | > 35 years old | |
| --- | --- | --- | --- | --- |
| Genes | **MDD** | **SCZ** | **MDD** | **SCZ** |
|  | **INFLAMMATION** | | | |
| *AIM2* | F=1.837; t=1.787; df=26; p=0.0857 | U=73; ranks:178,228; p=0.265 (exact) | U=85; ranks:190,188; p=0.793 (exact) | F=3.8; t=2.253; df=19.4; **p=0.036** |
| *ASC* | U=95; ranks:206,200; p=0.91(exact) | F=4.631; t=0.8574; df=18.36; p=0.4023 | U=97; ranks:202,204; p=0.982 (exact) | U=85; ranks:216,190; p=0.5714 (exact) |
| *BID* | U=81; ranks:186,220; p=0.454 (exact) | U=66; ranks:207,144; p=0.374 (exact) | U=87; ranks:192,214; p=0.635 (exact) | U=55; ranks:160,246; **p=0.0497** (exact) |
| *CASPASE 1* | U=89; ranks:184,194; p=0.943 (exact) | U=76; ranks:167,211; p=0.4879 (exact) | U=74; ranks:227,179; p=0.2852 (exact) | U=66; ranks:235,171; p=0.1499 (exact) |
| *CASPASE 2* | U=76; ranks:181,225; p=0.329 (exact) | U=75; ranks:198,153; p=0.667 (exact) | F=1.137; t=1.164; df=26; p=0.255 | F=6.612; t=2.316; df=16.84; **p=0.0335** |
| *CASPASE 4* | U=82; ranks:173,205; p=0.6848 (exact) | U=69; ranks:160,218; p=0.3022 (exact) | U=84; ranks:217,189; p=0.5409 (exact) | U=80; ranks:185,221; p=0.4274 (exact) |
| *CASPASE 5* | U=82; ranks:187,219; p=0.4824 (exact) | U=77; ranks:182,224; p=0.3519 (exact) | U=89; ranks:194,212; p=0.7006 (exact) | U=58; ranks:163,243; p=0.069 (exact) |
| *CASPASE 8* | U=85; ranks:190,216; p=0.571 (exact) | U=80; ranks:185,193; p=0.616 (exact) | U=83; ranks:188,218; p=0.511 (exact) | U=97; ranks:204,202; p=0.982 (exact) |
| *IL1β* | U=81; ranks:186,220; p=0.4544 (exact) | U=76; ranks:181,225; p=0.3287 (exact) | U=76; ranks:197,181; p=0.4879 (exact) | F=1.891; t=0.3369; df=25; p=0.739 |
| *IL18* | U=90; ranks:211,195; p=0.7345 (exact) | U=97; ranks:202,204; p=0.982 (exact) | U=79; ranks:222,184; p=0.4013 (exact) | U=83; ranks:218,188; p=0.5112 (exact) |
| *NALP1* | U=97; ranks:202,204; p=0.982 (exact) | U=63.09; ranks:181,225; p=0.329 (exact) | U=90; ranks:197,181; p=0.981 (exact) | F=2.091; t=0.956; df=26; p=0.348 |
| *NLRC4* | U=96; ranks:205,201; p=0.946 (exact) | F=194.5; t=1.022; df=13.13; p=0.3253 | U=61; ranks:166,212; p=0.155 (exact) | F=1.711; t=1.053; df=26; p=0.3021 |
| *NLRP3* | U=70; ranks:175,231; p=0.21 (exact) | F=3.718; t=0.7389; df=19.5; p=0.4687 | F=4.818; t=1.517; df=16.51; p=0.148 | F=4.864; t=1.235; df=18.13; p=0.2326 |
| *NRF2* | U=79; ranks:184,222; p=0.4013 (exact) | F=2.286; t=2.215; df=26; **p=0.0357** | U=92; ranks:209,197; p=0.8036 (exact) | U=91; ranks:196,210; p=0.769 (exact) |
| *TXNIP* | U=85; ranks:190,216; p=0.571 (exact) | U=73; ranks:178,173; p=0.595 (exact) | U=93; ranks:198,208; p=0.839 (exact) | U=68; ranks:173,233; p=0.178 (exact) |
|  | **AUTOPHAGY** | | | |
| *ATG3* | U=91; ranks:210,196; p=0.769 (exact) | F=2.08; t=1.163; df=26; p=0.255 | U=80; ranks:221,185; p=0.427 (exact) | F=2.055; t=1.702; df=26; p=0.1006 |
| *ATG7* | F=1.201; t=3.4; df=26; **p=0.0022** | F=1.505; t=0.402; df=26; p=0.691 | F=4.567; t=2.496; df=18.43; **p=0.0222** | F=5.069; t=1.665; df=17.94; p=0.113 |
| *ATG16L1* | F=1.296; t=1.129; df=25; p=0.27 | F=1.04; t=1.248; df=25; p=0.224 | F=1.687; t=1.734; df=26; p=0.0947 | F=1.023; t=1.152; df=26; p=0.26 |
| *BECLIN1* | F=1.78; t=0.4006; df=26; p=0.692 | F=2.349; t=1.651; df=26; p=0.1107 | F=1.373; t=1.402 df=26; p=0.1728 | F=2.188; t=0.00503; df=26; p=0.996 |
| *CYSTATIN C* | U=85; ranks:190,216; p=0.571 (exact) | U=54; ranks:159,247; **p=0.0444** (exact) | F=4.28; t=1.795; df=18.76; p=0.089 | U=85; ranks:190,216; p=0.571 (exact) |
| *GALECTIN 3* | U=74; ranks:179,227; p=0.2852 (exact) | U=67; ranks:172,234; p=0.1636 (exact) | U=88; ranks:213,193; p=0.6673 (exact) | U=70; ranks:175,231; p=0.21 (exact) |
| *GALECTIN 9* | F=1.292; t=0.512; df=26; p=0.613 | F=2.125; t=0.8941; df=26; p=0.3795 | F=1.154; t=1.554; df=26; p=0.1324 | F=1.921; t=1.198; df=26; p=0.2417 |
| *LAMP2A* | U=92; ranks:209,197; p=0.804 (exact) | U=95; ranks:206,200; p=0.91 (exact) | U=94; ranks:297,199; p=0.874 (exact) | U=58; ranks:243,163; p=0.069 (exact) |
| *LC3α* | F=1.482; t=0.338; df=25; p=0.738 | F=1.182; t=2.113; df=25; **p=0.0447** | F=2.311; t=1.318; df=26; p=0.199 | F=1.574; t=0.581; df=26; p=0.567 |
| *mTOR* | F=1.751; t=1.75; df=26; p=0.0918 | F=1.969; t=0.2022; df=26; p=0.8413 | F=1.126; t=0.11; df=26; p=0.9132 | F=2.061; t=1.218; df=26; p=0.2343 |
| *RAB5* | F=3.886; t=0.4055; df=19.28; p=0.6896 | U=60; ranks:241,165; p=0.0849 (exact) | F=1.541; t=0.532; df=26; p=0.5993 | F=1.684; t=1.392; df=26; p=0.1758 |
| *RAB7* | F=1.251; t=0.967; df=26; p=0.342 | F=1.12; t=0.182; df=26; p=0.857 | F=1.161; t=2.103; df=26; **p=0.0453** | U=80; ranks:185,221; p=0.427 (exact) |
| *SQSTM1* | F=2.558; t=0.7383; df=26; p=0.4669 | F=1.251; t=0.8159; df=26; p=0.422 | U=84; ranks:189,217; p=0.5409 (exact) | U=72; ranks:177,229; p=0.2456 (exact) |
| *ULK1* | F=1.792; t=1.47; df=26; p=0.1534 | F=1.197; t=1.298; df=26; p=0.2057 | F=1.069; t=0.0217; df=26; p=0.9829 | F=1.047; t=1.162; df=26; p=0.2556 |
|  | **UPR** | | | |
| *ATF4* | F=1.862; t=1.156; df=26; p=0.2583 | F=2.708; t=2.405; df=26; p=0.0236 | F=2.643; t=0.9339; df=26; p=0.359 | U=93; ranks:198,208; p=0.8388 (exact) |
| *ATF6* | U=94; ranks:199,207; p=0.8743 (exact) | U=80; ranks:185,221; p=0.4274 (exact) | U=87; ranks:192,214; p=0.6347 (exact) | F=1.817; t=0.7296; df=26; p=0.4722 |
| *BDNF* | U=90; ranks:211,195; p=0.7345 (exact) | U=52; ranks:221,157; p=0.0609 (exact) | F=3.455; t=1.113; df=19.94; p=0.279 | F=2.123; t=0.5128; df=26; p=0.6124 |
| *BiP* | U=65; ranks:236,170; p=0.1371 (exact) | U=76; ranks:225,181; p=0.3287 (exact) | F=4.836; t=0.1054; df=18.16; p=0.9172 | F=2.097; t=0.9219; df=26; p=0.3651 |
| *CHOP* | U=62; ranks:239,167; p=0.1035 (exact) | F=2.239; t=1.561; df=26; p=0.1305 | U=74; ranks:227,179; p=0.2852 (exact) | U=97; ranks:204,202; p=0.982 (exact) |
| *IF2α* | F=1.08; t=0.2651; df=26; p=0.793 | U=81; ranks:186,220; p=0.4544 (exact) | U=90; ranks:211,195; p=0.7345 (exact) | U=84; ranks:189,217; p=0.5409 (exact) |
| *IRE1α* | F=1.496; t=3.13; df=26; **p=0.0043** | F=2.196; t=3.287; df=26; **p=0.0029** | F=1.051; t=3.767; df=26; **p=0.0009** | F=3.525; t=3.544; df=26; **p=0.0015** |
| *PERK* | F=1.322; t=0.0759; df=26; p=0.9401 | F=3.191; t=0.576; df=20.42; p=0.5709 | U=64; ranks:169,237; p=0.1251 (exact) | U=98; ranks:203,203; p>0.9999 (exact) |
| *XBP1* | F=1.139; t=0.8765; df=26; p=0.3888 | F=3.493; t=0.6633; df=19.88; p=0.5148 | F=8.69; t=0.5962; df=15.95; p=0.5594 | U=91; ranks:210,196; p=0.7688 (exact) |
|  | **PROTEINS** | | | |
|  | **INFLAMMATION** | | | |
| AIM2 | U=40; ranks:261,145; **p=0.0067** (exact) | U=40; ranks:261,145; **p=0.0067** (exact) | F=1.959; t=2.603; df=25; **p=0.0153** | F=1.274; t=1.693; df=26; p=0.1024 |
| CASPASE 1 | U=55; ranks:146,232; p=0.085 (exact) | F=3.218; t=1.046; df=25; p=0.3057 | F=2.137; t=0.2252; df=25; p=0.8237 | F=2.316; t=1.018; df=25; p=0.3183 |
| CASPASE 5 | F=1.156; t=1.229; df=25; p=0.2306 | F=1.523; t=2.073; df=26; **p=0.0482** | U=67; ranks:220,158; p=0.2588 (exact) | U=32; ranks:255,123; **p=0.0033** (exact) |
| CASPASE 8 | F=1.091; t=0.2063; df=26; p=0.8382 | F=1.878; t=0.3855; df=26; p=0.7030 | F=2.724; t=0.1385; df=26; p=0.8909 | F=1.161; t=1.286; df=26; p=0.2096 |
| IL1β | F=1.141; t=0.71; df=24; p=0.4848 | F=1.497; t=1.9; df=25; p=0.069 | F=2.19; t=0.295; df=26; p=0.771 | F=2.241; t=0.5682; df=26; p=0.5747 |
| IL18 | U=84; ranks:175,176; p>0.999 (exact) | U=49; ranks:140,238; **p=0.0427** (exact) | U=87; ranks:214,192; p=0.635 (exact) | U=77; ranks:182,224; p=0.3519 (exact) |
| NLRC4 | F=2.926; t=1.915; df=25; p=0.067 | F=2.075; t=2.164; df=24; **p=0.0407** | F=1.115; t=1.61; df=26; p=0.1194 | F=1.016; t=1.24; df=26; p=0.2261 |
| NLRP3 | U=36; ranks:265,141; **p=0.0035** (exact) | U=17; ranks:284,122; **p<0.0001** (exact) | F=1.311; t=2.539; df=26; **p=0.0174** | F=1; t=4.072; df=26; **p=0.0004** |
| AUTOPHAGY | | | |  |
| ATG7 | U=41; ranks:260,146; **p=0.0079** (exact) | U=31; ranks:270,136; **p=0.0014** (exact) | F=2.031; t=3.384; df=26; **p=0.0023** | U=18; ranks:283,123; **p<0.0001** (exact) |
| mTOR | U=30; ranks:271,135; **p=0.0012** (exact) | U=39; ranks:262,144; **p=0.0058** (exact) | F=1.524; t=2.384; df=26; **p=0.0247** | F=2.704; t=3.402; df=26; **p=0.0022** |
| RAB5A | F=1.865; t=2.64; df=26; **p=0.0138** | U=20; ranks:281,125; **p=0.0001** (exact) | F=1.615; t=4.025; df=26; **p=0.0004** | F=1.957; t=2.811; df=26; **p=0.0093** |
| RAB7 | F=1.233; t=1.213; df=26; p=0.2361 | F=1.983; t=3.391; df=26; **p=0.0022** | F=5.651; t=1.417; df=17.46; p=0.1742 | F=5.177; t=0.4159; df=17.84; p=0.6824 |
| UPR | | | | |
| IRE1α | U=18; ranks:255,123; **p=0.0002** (exact) | F=2.259; t=8.521; df=25; **p<0.0001** | F=1.683; t=5.569; df=26; **p<0.0001** | F=1.733; t=11.37; df=26; **p<0.0001** |
| PROTEASOME 26S | F=1.405; t=1.68; df=24; p=0.1059 | F=1.442; t=1.63; df=25; p=0.1157 | F=1.417; t=0.631; df=26; p=0.534 | F=1.597; t=0.9369; df=26; p=0.3574 |

This table exhibits the analysis performed using Student’s *t*-test or the Mann-Whitney test when stratifying by age all genes and proteins considered.

**Table S4. Statistically significant differences in cause of death of SCZ individuals at the mRNA and protein levels.**

| GENES/PROTEINS | Cause of death in SCZ |
| --- | --- |
|  | **Natural vs Suicide** |
| INFLAMMATION | |
| *CASPASE 5 (g)* | U=51; ranks:117,289; **p=0.0469** (exact) |
| CASPASE 5 (p) | F=1.266; t=1.859; df=25; p=0.0749 |
| CASPASE 8 (p) | F=2.1; t=1.882; df=26; **p=0.0109** |
| *IL18 (g)* | F=1.651; t=2.722; df=26; **p=0.0114** |
| AUTOPHAGY | |
| *SQSTM1* *(g)* | F=1.233; t=4.047; df=26; **p=0.0004** |
| UPR | |
| *IRE1α* *(g)* | F=2.19; t=3.876; df=25; **p=0.0006** |

This table presents the analysis performed using Student’s *t*-test or the Mann-Whitney test in SCZ subjects that died by suicide, in contrast to those who died of natural causes. *(g)* and (p) stand for ‘gene’ and ‘protein’ respectively.

**Table S5. Spearman correlation analysis between gene and protein expression levels.**

|  | Control | | MDD | | SCZ | |
| --- | --- | --- | --- | --- | --- | --- |
|  | **Coefficient** | ***p* value** | **Coefficient** | ***p* value** | **Coefficient** | ***p* value** |
| AIM2 | 0.002 | 0.991 | 0.284 | 0.150 | 0.257 | 0.186 |
| CASPASE 1 | 0.162 | 0.410 | 0.201 | 0.304 | -0.081 | 0.682 |
| CASPASE 5 | -0.205 | 0.295 | -0.304 | 0.116 | 0.027 | 0.890 |
| CASPASE 8 | 0.352 | 0.066 | -0.150 | 0.446 | 0.092 | 0.647 |
| IL1β | 0.092 | 0.642 | -0.226 | 0.247 | 0.324 | 0.093 |
| IL18 | -0.050 | 0.801 | -0.043 | 0.829 | 0.312 | 0.106 |
| NLRC4 | -0.302 | 0.119 | -0.320 | 0.104 | 0.371 | 0.052 |
| NLRP3 | -0.013 | 0.949 | -0.328 | 0.094 | -0.028 | 0.886 |
| ATG7 | -0.148 | 0.453 | -0.105 | 0.595 | 0.091 | 0.646 |
| mTOR | -0.248 | 0.203 | -0.048 | 0.808 | -0.019 | 0.923 |
| RAB5A | 0.047 | 0.812 | -0.354 | 0.065 | -0.090 | 0.650 |
| RAB7 | -0.314 | 0.104 | -0.058 | 0.769 | -0.223 | 0.253 |
| IRE1α | -0.064 | 0.746 | 0.119 | 0.547 | 0.319 | 0.099 |

Spearman correlation coefficients and corresponding *p* values are shown for each marker analyzed at both mRNA and protein levels in control, MDD, and SCZ.

**Table S6. Demographic, clinical, and toxicological data of controls, MDD, and SCZ subjects.**

|  | Diagnostic | Sex (M/F) | Age (years) | PMI (h) | Cause of death | Drug blood levels |
| --- | --- | --- | --- | --- | --- | --- |
| 1 | Control | F | 50 | 11 | Natural | - |
|  | MDD | F | 50 | 13 | Natural | AD-free |
|  | SCZ | F | 53 | 17 | Natural | AP-free |
| 2 | Control | F | 57 | 14 | Natural | - |
|  | MDD | F | 58 | 27 | Natural | AD-free |
|  | SCZ | F | 59 | 19 | Natural | AP-free |
| 3 | Control | F | 41 | 14 | Natural | - |
|  | MDD | F | 37 | 12 | Natural | AD-free |
|  | SCZ | F | 38 | 19 | Suicide | Clotiapine, Zuclopentixol,  BZD |
| 4 | Control | M | 51 | 18 | Accident | - |
|  | MDD | M | 51 | 24 | Suicide | AD-free |
|  | SCZ | M | 52 | 16 | Suicide | AP-free |
| 5 | Control | M | 34 | 16 | Accident | - |
|  | MDD | M | 34 | 18 | Suicide | AD-free |
|  | SCZ | M | 35 | 11 | Natural | Clozapine,  BZD |
| 6 | Control | M | 37 | 11 | Accident | - |
|  | MDD | M | 39 | 18 | Suicide | AD-free |
|  | SCZ | M | 37 | 18 | Suicide | Olanzapine,  BZD |
| 7 | Control | M | 58 | 16 | Accident | - |
|  | MDD | M | 59 | 31 | Suicide | AD-free |
|  | SCZ | M | 60 | 17 | Natural | AP-free |
| 8 | Control | M | 61 | 23 | Accident | - |
|  | MDD | M | 61 | 43 | Suicide | AD-free |
|  | SCZ | M | 62 | 10 | Natural | Tioridazine |
| 9 | Control | M | 71 | 21 | Natural | - |
|  | MDD | M | 72 | 20 | Suicide | AD-free |
|  | SCZ | M | 72 | 14 | Natural | Periciazine |
| 10 | Control | F | 32 | 19 | Natural | - |
|  | MDD | F | 30 | 21 | Suicide | Citalopram,  Levomepromazine,  BZD |
|  | SCZ | F | 30 | 17 | Suicide | Haloperidol,  Trazodone,  BZD |
| 11 | Control | M | 37 | 21 | Accident | - |
|  | MDD | M | 37 | 20 | Suicide | Fluoxetine,  Olanzapine,  BZD |
|  | SCZ | M | 37 | 11 | Suicide | Olanzapine,  BZD |
| 12 | Control | M | 23 | 4 | Accident | - |
|  | MDD | M | 23 | 11 | Suicide | Mirtazapine,  BZD |
|  | SCZ | M | 23 | 13 | Suicide | AP-free |
| 13 | Control | F | 35 | 17 | Accident | - |
|  | MDD | F | 34 | 6 | Suicide | Venlafaxine,  Citalopram,  Amisulpiride,  Topiramate,  Clozapine, BZD |
|  | SCZ | F | 35 | 3 | Suicide | Quetiapine,  BZD |
| 14 | Control | F | 42 | 37 | Accident | - |
|  | MDD | F | 43 | 4 | Natural | Fluoxetine,  BZD |
|  | SCZ | F | 42 | 14 | Suicide | Clozapine,  Quetiapine,  Sulpiride,  BZD |
| 15 | Control | M | 32 | 4 | Accident | - |
|  | MDD | M | 33 | 38 | Suicide | AD-free |
|  | SCZ | M | 33 | 14 | Suicide | AP-free |
| 16 | Control | F | 33 | 24 | Accident | - |
|  | MDD | F | 32 | 15 | Suicide | AD-free |
|  | SCZ | F | 33 | 22 | Natural | Olanzapine,  Levomepromazine,  Citalopram,  BZD |
| 17 | Control | M | 28 | 15 | Accident | - |
|  | MDD | M | 27 | 14 | Suicide | AD-free |
|  | SCZ | M | 25 | 16 | Suicide | AP-free |
| 18 | Control | M | 29 | 17 | Accident | - |
|  | MDD | M | 30 | 4 | Suicide | AD-free |
|  | SCZ | M | 28 | 13 | Suicide | AP-free |
| 19 | Control | M | 25 | 18 | Accident | - |
|  | MDD | M | 24 | 37 | Suicide | AD-free |
|  | SCZ | M | 26 | 21 | Suicide | AP-free |
| 20 | Control | M | 33 | 23 | Accident | - |
|  | MDD | M | 33 | 32 | Natural | AD-free |
|  | SCZ | M | 33 | 20 | Suicide | Quetiapine,  BZD |
| 21 | Control | M | 36 | 33 | Accident | - |
|  | MDD | M | 35 | 20 | Suicide | Fluoxetine,  BZD |
|  | SCZ | M | 36 | 8 | Suicide | Olanzapine |
| 22 | Control | M | 33 | 17 | Accident | - |
|  | MDD | M | 35 | 13 | Suicide | AD-free |
|  | SCZ | M | 35 | 42 | Natural | AP-free |
| 23 | Control | M | 26 | 5 | Accident | - |
|  | MDD | M | 28 | 5 | Suicide | AD-free |
|  | SCZ | M | 26 | 15 | Suicide | Olanzapine |
| 24 | Control | F | 23 | 18 | Accident | - |
|  | MDD | F | 26 | 26 | Suicide | AD-free |
|  | SCZ | F | 25 | 19 | Suicide | AP-free |
| 25 | Control | M | 57 | 3 | Accident | - |
|  | MDD | M | 56 | 3 | Natural | AD-free |
|  | SCZ | M | 58 | 6 | Natural | Clotiapine |
| 26 | Control | M | 50 | 2 | Natural | - |
|  | MDD | M | 49 | 26 | Suicide | AD-free |
|  | SCZ | M | 51 | 18 | Natural | Paliperidone,  BZD |
| 27 | Control | F | 66 | 16 | Natural | - |
|  | MDD | F | 66 | 19 | Suicide | AD-free |
|  | SCZ | F | 67 | 22 | Natural | AP-free |
| 28 | Control | M | 32 | 16 | Accident | - |
|  | MDD | M | 32 | 16 | Suicide | AD-free |
|  | SCZ | M | 32 | 21 | Suicide | AP-free |

AD, antidepressant; AP, antipsychotic; PMI, postmortem interval or time elapsed between death and autopsy; BZD, benzodiazepines.

**Table S7. Fluorescent probes employed to target autophagy, inflammatory, and UPR-related gene expression.**

| Genes | Fluorescent tag |
| --- | --- |
| *ATG3* | HEX |
| *ATG7* | Texas Red |
| *ATG16L1* | FAM |
| *BECLIN1* | Cy5 |
| *CYSTATIN C* | FAM |
| *GALECTIN 3* | Cy5 |
| *GALECTIN 9* | FAM |
| *LAMP2A* | Cy5 |
| *LC3α* | Cy5 |
| *mTOR* | FAM |
| *RAB5* | Texas Red |
| *RAB7* | Cy5 |
| *SQSTM1* | HEX |
| *ULK1* | HEX |
| *AIM2* | Cy5 |
| *ASC* | FAM |
| *BID* | FAM |
| *CASPASE 1* | HEX |
| *CASPASE 2* | HEX |
| *CASPASE 4* | HEX |
| *CASPASE 5* | Texas Red |
| *CASPASE 8* | Cy5 |
| *IL1β* | Texas Red |
| *IL18* | Cy5 |
| *NALP1* | Texas Red |
| *NLRC4* | HEX |
| *NLRP3* | FAM |
| *NRF2* | Cy5 |
| *TXNIP* | Texas Red |
| *ATF4* | Texas Red |
| *ATF6* | HEX |
| *BDNF* | HEX |
| *BiP* | FAM |
| *CHOP* | Cy5 |
| *IF2α* | FAM |
| *IRE1α* | Texas Red |
| *PERK* | Cy5 |
| *XBP1* | FAM |

**Table S8. List of primary and secondary antibodies used for Western blot analyses.**

| Proteins (MW) | Catalogue | Primary Antibody | Secondary Antibody |
| --- | --- | --- | --- |
| AIM2 (42 KDa) | Cell Signaling Technology, 12948 | 1:1000, 2.5% BSA in TBSt | Anti-Rabbit 1:2000 (Santa Cruz Biotechnology, sc-2357) in TBSt |
| ATG7  (78 kDa) | Cell Signaling Technology, 8558 | 1:1000, 5% BSA in TBSt |  |
| IRE1α (110 KDa) | Abcam, ab37073 | 1:1000, 2.5% BSA in TBSt |  |
| mTOR (289 kDa) | Cell Signaling Technology, 5536 | 1:1000, 5% BSA in TBSt |  |
| NLRC4 (110 KDa) | Cell Signaling Technology,12421 | 1:1000, 2.5% BSA in TBSt |  |
| NLRP3 (118 KDa) | Abcam, ab263899 |  |  |
| RAB5A (25 KDa) | Fisher Scientific, 17160752 |  | Anti-mouse 1:2000 (Santa Cruz Biotechnology, sc-516102) in TBSt |
| RAB7  (22 kDa) | Santa Cruz Biotechnology, sc-376362 | 1:1000 in TBSt |  |
| β-ACTIN  (42 kDa) | Santa Cruz Biotechnology, sc-47778 | 1:10000 in TBSt | - |

This table includes the target protein with its molecular weight (MW), supplier, catalogue number, dilution, and incubation conditions. Antibodies were diluted in blocking solution containing BSA. BSA: bovine serum albumin; TBSt: Tris-buffered saline supplemented with Tween-20.

**Table S9. Data distribution of inflammatory, autophagy, and UPR genes and proteins in the regression models of this study.**

|  | CT | | | | | MDD | | | | | SCZ | | | | |
| --- | --- | --- | --- | --- | --- | --- | --- | --- | --- | --- | --- | --- | --- | --- | --- |
| GENES | **Mean** | **SD** | **Median** | **Min** | **Max** | **Mean** | **SD** | **Median** | **Min** | **Max** | **Mean** | **SD** | **Median** | **Min** | **Max** |
| INFLAMMATION | | | | | | | | | | | | | | | |
| *AIM2* | 19.24 | 5.28 | 18.90 | 9.17 | 29.82 | 24.28 | 14.20 | 21.52 | 5.01 | 79.43 | 26.38 | 14.36 | 21.12 | 10.06 | 80.09 |
| *ASC* | 12.56 | 4.59 | 11.30 | 5.05 | 21.32 | 13.82 | 8.97 | 11.73 | 5.82 | 44.15 | 14.27 | 10.07 | 12.65 | 3.28 | 50.40 |
| *BID* | 10.29 | 6.05 | 9.32 | 1.04 | 32.47 | 11.03 | 4.66 | 10.57 | 5.12 | 31.41 | 16.18 | 22.51 | 11.61 | 0.47 | 118.74 |
| *CASPASE 1* | 60.08 | 234.69 | 13.07 | 5.43 | 1256.76 | 15.33 | 10.91 | 12.90 | 4.41 | 45.96 | 15.62 | 11.62 | 12.43 | 2.59 | 53.88 |
| *CASPASE 2* | 5.27 | 2.65 | 4.67 | 1.17 | 15.90 | 5.96 | 2.27 | 5.65 | 2.46 | 15.12 | 8.66 | 12.12 | 5.19 | 0.12 | 65.65 |
| *CASPASE 4* | 58.16 | 235.06 | 11.87 | 4.88 | 1256.76 | 15.84 | 14.69 | 10.02 | 4.38 | 74.62 | 19.87 | 17.38 | 14.27 | 3.13 | 83.57 |
| *CASPASE 5* | 45.39 | 71.43 | 30.11 | 4.17 | 393.19 | 86.99 | 196.14 | 36.77 | 3.49 | 1053.23 | 76.74 | 88.50 | 46.09 | 2.80 | 384.36 |
| *CASPASE 8* | 6.78 | 6.57 | 3.96 | 0.86 | 23.24 | 13.45 | 19.56 | 6.94 | 0.18 | 86.60 | 10.19 | 13.62 | 6.18 | 0.07 | 65.89 |
| *IL1β* | 75.03 | 182.07 | 37.42 | 10.75 | 992.21 | 197.21 | 494.10 | 30.31 | 7.07 | 2201.89 | 68.03 | 106.67 | 36.34 | 8.53 | 524.90 |
| *IL18* | 34.16 | 16.55 | 29.54 | 14.57 | 82.29 | 35.11 | 26.24 | 28.80 | 12.83 | 121.98 | 37.80 | 29.95 | 29.55 | 10.90 | 126.90 |
| *NALP1* | 70.45 | 40.73 | 59.76 | 23.45 | 223.38 | 81.21 | 70.18 | 62.72 | 27.67 | 314.11 | 81.29 | 40.62 | 78.84 | 23.99 | 195.26 |
| *NLRC4* | 105.49 | 226.01 | 63.98 | 41.93 | 1256.76 | 70.11 | 25.90 | 68.57 | 35.29 | 178.94 | 64.78 | 19.60 | 65.40 | 11.68 | 99.63 |
| *NLRP3* | 94.41 | 26.24 | 94.21 | 46.69 | 154.25 | 119.12 | 50.53 | 106.87 | 47.48 | 282.10 | 109.54 | 52.25 | 106.60 | 30.78 | 244.81 |
| *NRF2* | 640.30 | 262.95 | 573.87 | 260.38 | 1631.20 | 670.44 | 302.92 | 605.94 | 271.53 | 1906.22 | 743.12 | 256.03 | 730.87 | 319.90 | 1315.66 |
| *TXNIP* | 44.14 | 36.63 | 29.48 | 7.68 | 154.75 | 49.14 | 39.54 | 32.14 | 10.96 | 163.16 | 117.31 | 279.40 | 46.53 | 3.30 | 1485.40 |
| AUTOPHAGY | | | | | | | | | | | | | | | |
| *ATG3* | 100.78 | 24.27 | 97.56 | 59.19 | 164.78 | 97.46 | 25.18 | 90.92 | 59.33 | 177.18 | 98.61 | 25.42 | 93.35 | 57.89 | 164.78 |
| *ATG7* | 149.12 | 21.02 | 151.72 | 116.18 | 231.57 | 178.11 | 30.42 | 177.78 | 93.96 | 234.54 | 152.79 | 35.28 | 167.26 | 70.24 | 231.57 |
| *ATG16L1* | 12.10 | 2.60 | 11.76 | 7.80 | 16.57 | 13.50 | 2.64 | 13.54 | 6.57 | 18.41 | 12.32 | 2.65 | 11.66 | 3.00 | 16.57 |
| *BECLIN1* | 24.66 | 5.13 | 23.95 | 13.42 | 32.57 | 25.71 | 6.03 | 25.20 | 12.51 | 36.81 | 24.46 | 7.26 | 24.64 | 8.24 | 32.57 |
| *CYSTATIN C* | 639.35 | 291.31 | 643.42 | 57.83 | 3201.13 | 675.84 | 577.59 | 514.38 | 173.15 | 3242.71 | 649.54 | 610.88 | 768.84 | 171.19 | 3201.13 |
| *GALECTIN 3* | 62.68 | 22.51 | 60.69 | 21.16 | 283.26 | 74.17 | 45.48 | 66.47 | 23.32 | 235.29 | 70.24 | 60.95 | 71.54 | 7.81 | 283.26 |
| *GALECTIN 9* | 24.89 | 6.00 | 25.41 | 11.74 | 37.52 | 27.14 | 5.65 | 26.44 | 14.50 | 41.37 | 25.02 | 8.45 | 24.60 | 4.86 | 37.52 |
| *LAMP2A* | 100.11 | 62.52 | 82.51 | 37.31 | 326.03 | 90.35 | 39.81 | 83.90 | 39.59 | 212.60 | 99.37 | 61.64 | 77.13 | 40.21 | 326.03 |
| *LC3α* | 61.43 | 13.22 | 58.32 | 41.93 | 80.79 | 66.29 | 17.28 | 66.74 | 28.14 | 101.53 | 61.17 | 14.93 | 52.82 | 24.10 | 80.79 |
| *mTOR* | 53.43 | 9.77 | 53.87 | 38.85 | 78.04 | 57.22 | 12.65 | 54.72 | 26.79 | 86.13 | 54.81 | 11.05 | 55.90 | 29.73 | 78.04 |
| *RAB5* | 89.26 | 11.70 | 88.96 | 68.13 | 101.54 | 89.01 | 18.59 | 92.09 | 51.21 | 139.06 | 88.56 | 20.12 | 85.00 | 10.26 | 101.54 |
| *RAB7* | 363.52 | 54.11 | 350.14 | 279.03 | 493.16 | 395.25 | 59.19 | 383.23 | 306.71 | 544.83 | 362.82 | 68.56 | 385.29 | 158.58 | 493.16 |
| *SQSTM1* | 94.42 | 25.65 | 90.12 | 48.82 | 141.61 | 97.80 | 19.90 | 97.78 | 61.10 | 138.97 | 95.41 | 19.30 | 100.14 | 67.71 | 141.61 |
| *ULK1* | 18.77 | 5.48 | 18.27 | 5.69 | 29.76 | 20.38 | 6.33 | 19.14 | 3.81 | 37.31 | 19.16 | 6.23 | 19.55 | 6.93 | 29.76 |
| UPR | | | | | | | | | | | | | | | |
| *ATF4* | 151.94 | 26.43 | 149.94 | 106.74 | 217.92 | 149.58 | 32.67 | 149.49 | 88.59 | 210.07 | 154.25 | 104.57 | 138.27 | 36.63 | 655.49 |
| *ATF6* | 362.42 | 178.60 | 329.87 | 237.72 | 1256.76 | 337.65 | 63.26 | 337.42 | 183.04 | 462.03 | 339.82 | 66.24 | 346.18 | 114.50 | 459.48 |
| *BDNF* | 125.81 | 223.16 | 81.02 | 31.54 | 1256.76 | 89.25 | 42.08 | 92.26 | 13.17 | 174.42 | 68.19 | 36.31 | 64.20 | 7.34 | 158.52 |
| *BiP* | 111.54 | 21.09 | 112.60 | 74.39 | 163.22 | 106.40 | 35.05 | 106.30 | 20.49 | 186.28 | 101.00 | 26.95 | 99.22 | 29.40 | 139.73 |
| *CHOP* | 31.20 | 13.22 | 27.32 | 18.72 | 90.75 | 27.64 | 12.25 | 25.48 | 12.72 | 66.65 | 29.88 | 13.31 | 25.31 | 6.37 | 76.69 |
| *IF2α* | 878.77 | 332.68 | 796.33 | 420.62 | 2027.06 | 923.86 | 521.93 | 756.88 | 485.82 | 3091.51 | 1018.94 | 544.70 | 958.68 | 574.39 | 3408.00 |
| *IRE1α* | 98.49 | 18.71 | 96.77 | 62.37 | 135.98 | 123.27 | 20.75 | 126.28 | 84.57 | 180.91 | 130.94 | 30.07 | 132.68 | 73.58 | 181.11 |
| *PERK* | 136.75 | 25.45 | 130.74 | 90.42 | 223.04 | 139.63 | 24.50 | 143.99 | 70.44 | 169.41 | 132.01 | 34.98 | 132.29 | 40.12 | 208.02 |
| *XBP1* | 246.87 | 44.71 | 236.25 | 175.75 | 348.78 | 248.05 | 87.67 | 244.82 | 75.53 | 556.10 | 257.51 | 178.85 | 232.36 | 51.74 | 1063.55 |
| PROTEINS | | | | | | | | | | | | | | | |
| INFLAMMATION | | | | | | | | | | | | | | | |
| AIM2 | 95.73 | 18.51 | 96.62 | 51.89 | 140.01 | 74.26 | 22.22 | 77.29 | 2.41 | 113.45 | 74.97 | 25.59 | 67.40 | 29.46 | 127.99 |
| CASPASE 1 | 33.86 | 16.74 | 31.22 | 15.95 | 106.33 | 33.21 | 8.32 | 31.70 | 19.29 | 55.85 | 33.74 | 11.25 | 32.62 | 17.37 | 53.80 |
| CASPASE 5 | 216.53 | 101.17 | 208.29 | 51.06 | 509.21 | 160.22 | 112.32 | 192.59 | 0.00 | 424.05 | 120.01 | 80.53 | 98.67 | 0.00 | 277.04 |
| CASPASE 8 | 2.57 | 1.17 | 2.43 | 0.47 | 5.65 | 2.54 | 1.41 | 2.46 | 0.29 | 5.64 | 2.48 | 1.03 | 2.25 | 0.97 | 5.23 |
| IL1β | 42.20 | 22.21 | 37.90 | 21.74 | 141.53 | 117.31 | 279.40 | 46.53 | 3.30 | 1485.40 | 44.43 | 13.58 | 45.40 | 24.26 | 67.64 |
| IL18 | 2.15 | 1.09 | 1.94 | 1.05 | 6.92 | 36.99 | 17.06 | 33.27 | 17.29 | 111.16 | 2.31 | 0.81 | 2.13 | 1.13 | 4.22 |
| NLRC4 | 105.11 | 60.27 | 94.85 | 46.60 | 385.25 | 74.85 | 32.57 | 64.34 | 32.33 | 146.27 | 85.24 | 50.56 | 74.28 | 32.10 | 296.69 |
| NLRP3 | 102.82 | 25.78 | 96.61 | 66.99 | 184.16 | 76.76 | 22.62 | 75.50 | 40.27 | 113.57 | 66.14 | 21.53 | 72.14 | 25.23 | 103.16 |
| AUTOPHAGY | | | | | | | | | | | | | | | |
| ATG7 | 102.75 | 12.47 | 102.31 | 74.98 | 141.20 | 82.16 | 20.47 | 78.01 | 48.25 | 122.35 | 77.51 | 17.26 | 77.85 | 46.15 | 108.84 |
| mTOR | 94.48 | 15.61 | 98.46 | 52.23 | 121.95 | 73.70 | 20.50 | 70.46 | 32.79 | 109.03 | 66.95 | 26.99 | 60.32 | 25.79 | 125.25 |
| RAB5A | 96.75 | 16.21 | 95.89 | 64.58 | 129.49 | 72.73 | 21.92 | 67.09 | 36.08 | 134.49 | 69.58 | 21.31 | 69.74 | 25.81 | 117.61 |
| RAB7 | 95.64 | 19.10 | 94.27 | 62.45 | 149.76 | 83.15 | 29.60 | 81.15 | 39.03 | 161.15 | 80.77 | 26.24 | 75.02 | 33.14 | 151.33 |
| UPR | | | | | | | | | | | | | | | |
| IRE1α | 99.15 | 26.36 | 97.03 | 60.98 | 207.09 | 58.00 | 21.81 | 53.18 | 30.22 | 115.07 | 43.23 | 11.33 | 42.96 | 22.42 | 65.54 |
| PROTEASOME 26S | 32.34 | 20.56 | 25.02 | 9.32 | 109.38 | 2.05 | 1.24 | 1.78 | 0.85 | 7.41 | 42.29 | 21.68 | 43.69 | 12.30 | 89.03 |

These measures summarize central tendency, variability, and range by the mean, standard deviation (SD), median, minimum (Min), and maximum (Max).

**Table S10. FDR-adjusted *q* values for gene and protein expression analyses in MDD, SCZ, and control subjects.**

|  | MDD | | | | SCZ | | | | CT |  |  |  |
| --- | --- | --- | --- | --- | --- | --- | --- | --- | --- | --- | --- | --- |
|  | **Tables**  **1 and 2** | **Tables**  **3 and 4** | **Tables**  **3 and 4** | **Tables**  **5 and 6** | **Tables**  **1 and 2** | **Tables**  **3 and 4** | **Tables**  **3 and 4** | **Tables**  **5 and 6** | **Tables**  **5 and 6** |  |  |  |
|  | **Logistic regression model** | **Sex stratification men** | **Sex stratification women** | **Age stratification** | **Logistic regression model** | **Sex stratification**  **men** | **Sex stratification women** | **Age stratification** | **Age stratification** |  |  |  |
|  | ***q* values** | | | | | | | | |  |  |  |
| GENES | | | | | | | | | |  |  |  |
| INFLAMMATION | | | | | | | | | |  |  |  |
| *AIM2* | 0.716 | 0.557 | 0.982 | 0.815 | 0.409 | 0.720 | 0.396 | 0.868 | 0.679 |  |  |  |
| *ASC* | 0.811 | 0.775 | 0.982 | 0.815 | 0.724 | 0.720 | 0.537 | 0.722 | 0.737 |  |  |  |
| *BID* | 0.811 | 0.775 | 0.982 | 0.777 | 0.544 | 0.819 | 0.552 | 0.726 | 0.380 |  |  |  |
| *CASPASE 1* | 0.828 | 0.960 | 0.982 | 0.777 | 0.864 | 0.819 | 0.537 | 0.697 | 0.450 |  |  |  |
| *CASPASE 2* | 0.785 | 0.521 | 0.982 | 0.913 | 0.544 | 0.720 | 0.552 | 0.726 | 0.679 |  |  |  |
| *CASPASE 4* | 0.811 | 0.787 | 0.982 | 0.783 | 0.774 | 0.720 | 0.715 | 0.726 | 0.450 |  |  |  |
| *CASPASE 5* | 0.811 | 0.775 | 0.982 | 0.779 | 0.523 | 0.720 | 0.552 | 0.726 | 0.679 |  |  |  |
| *CASPASE 8* | 0.722 | 0.742 | 0.982 | 0.777 | 0.544 | 0.720 | 0.692 | 0.799 | 0.498 |  |  |  |
| *IL1β* | 0.786 | 0.787 | 0.982 | 0.777 | 0.932 | 0.976 | 0.396 | 0.722 | 0.679 |  |  |  |
| *IL18* | 0.917 | 0.787 | 0.982 | 0.848 | 0.774 | 0.720 | 0.537 | 0.697 | 0.680 |  |  |  |
| *NALP1* | 0.812 | 0.787 | 0.982 | 0.777 | 0.612 | 0.720 | 0.707 | 0.697 | 0.450 |  |  |  |
| *NLRC4* | 0.811 | 0.787 | 0.982 | 0.848 | 0.774 | 0.976 | 0.692 | 0.726 | 0.450 |  |  |  |
| *NLRP3* | 0.454 | 0.466 | 0.982 | 0.777 | 0.523 | 0.720 | 0.722 | 0.726 | 0.641 |  |  |  |
| *NRF2* | 0.828 | 0.861 | 0.982 | 0.783 | 0.518 | 0.727 | 0.396 | 0.933 | 0.342 |  |  |  |
| *TXNIP* | 0.811 | 0.775 | 0.982 | 0.777 | 0.518 | 0.819 | 0.445 | 0.726 | 0.450 |  |  |  |
| AUTOPHAGY | | | | | | | | | |  |  |  |
| *ATG3* | 0.811 | 0.861 | 0.982 | 0.777 | 0.873 | 0.836 | 0.993 | 0.726 | 0.394 |  |  |  |
| *ATG7* | **0.023** | 0.114 | 0.982 | 0.777 | 0.518 | 0.720 | 0.429 | 0.726 | 0.679 |  |  |  |
| *ATG16L1* | 0.454 | 0.513 | 0.982 | 0.777 | 0.864 | 0.805 | 0.993 | 0.722 | 0.860 |  |  |  |
| *BECLIN1* | 0.811 | 0.566 | 0.982 | 0.777 | 0.544 | 0.976 | 0.396 | 0.943 | 0.342 |  |  |  |
| *CYSTATIN C* | 0.828 | 0.861 | 0.982 | 0.777 | 0.409 | 0.720 | 0.429 | 0.799 | 0.860 |  |  |  |
| *GALECTIN 3* | 0.785 | 0.742 | 0.982 | 0.777 | 0.409 | 0.720 | 0.435 | 0.726 | 0.860 |  |  |  |
| *GALECTIN 9* | 0.742 | 0.557 | 0.982 | 0.777 | 0.942 | 0.819 | 0.692 | 0.799 | 0.450 |  |  |  |
| *LAMP2A* | 0.811 | 0.775 | 0.982 | 0.777 | 0.774 | 0.727 | 0.903 | 0.726 | 0.34 |  |  |  |
| *LC3α* | 0.785 | 0.513 | 0.982 | 0.777 | 0.409 | 0.720 | 0.396 | 0.876 | 0.450 |  |  |  |
| *mTOR* | 0.785 | 0.719 | 0.982 | 0.777 | 0.544 | 0.720 | 0.715 | 0.697 | 0.860 |  |  |  |
| *RAB5* | 0.951 | 0.787 | 0.982 | 0.777 | 0.409 | 0.720 | 0.396 | 0.726 | 0.575 |  |  |  |
| *RAB7* | 0.454 | 0.114 | 0.982 | 0.827 | 0.876 | 0.720 | 0.552 | 0.726 | 0.659 |  |  |  |
| *SQSTM1* | 0.811 | 0.903 | 0.982 | 0.608 | 0.724 | 0.976 | 0.435 | **0.038** | 0.394 |  |  |  |
| *ULK1* | 0.785 | 0.775 | 0.982 | 0.777 | 0.942 | 0.836 | 0.692 | 0.228 | 0.860 |  |  |  |
| UPR | | | | | | | | | |  |  | 0.982 |
| *ATF4* | 0.828 | 0.787 | 0.982 | 0.777 | 0.942 | 0.727 | 0.396 | 0.943 | 0.380 |  |  |  |
| *ATF6* | 0.811 | 0.787 | 0.982 | 0.783 | 0.774 | 0.720 | 0.692 | 0.943 | 0.450 |  |  |  |
| *BDNF* | 0.811 | 0.787 | 0.982 | 0.777 | 0.429 | 0.720 | 0.396 | 0.943 | 0.394 |  |  |  |
| *BiP* | 0.811 | 0.782 | 0.982 | 0.777 | 0.518 | 0.720 | 0.396 | 0.943 | 0.460 |  |  |  |
| *CHOP* | 0.785 | 0.775 | 0.982 | 0.957 | 0.864 | 0.819 | 0.993 | 0.722 | 0.213 |  |  |  |
| *IF2α* | 0.828 | 0.861 | 0.982 | 0.777 | 0.544 | 0.727 | 0.445 | 0.726 | 0.394 |  |  |  |
| *IRE1α* | **0.023** | 0.114 | 0.982 | 0.777 | **0.019** | 0.114 | 0.396 | 0.726 | 0.679 |  |  |  |
| *PERK* | 0.828 | 0.787 | 0.982 | 0.777 | 0.774 | 0.976 | 0.396 | 0.722 | 0.394 |  |  |  |
| *XBP1* | 0.951 | 0.906 | 0.982 | 0.777 | 0.874 | 0.720 | 0.396 | 0.809 | 0.740 |  |  |  |
| PROTEINS | | | | | | | | | |  |  |  |
| INFLAMMATION | | | | | | | | |  |  |  |  |
| AIM2 | **0.004** | **0.016** | 0.299 | 0.958 | **0.007** | **0.019** | 0.290 | 0.440 | 0.787 |  |  |  |
| CASPASE 1 | 0.918 | 0.954 | 0.584 | 0.958 | 0.975 | 0.689 | 0.508 | 0.557 | 0.787 |  |  |  |
| CASPASE 5 | 0.109 | 0.191 | 0.299 | 0.958 | **0.003** | **0.012** | 0.290 | 0.440 | 0.787 |  |  |  |
| CASPASE 8 | 0.932 | 0.573 | 0.364 | 0.958 | 0.795 | 0.802 | 0.867 | 0.266 | 0.787 |  |  |  |
| IL1β | 0.868 | 0.068 | 0.757 | 0.958 | 0.663 | 0.526 | 0.859 | 0.378 | 0.787 |  |  |  |
| IL18 | 0.868 | 0.605 | 0.613 | 0.958 | 0.143 | 0.303 | 0.324 | 0.182 | 0.787 |  |  |  |
| NLRC4 | **0.036** | 0.122 | 0.291 | 0.958 | 0.309 | 0.463 | 0.322 | 0.981 | 0.787 |  |  |  |
| NLRP3 | **0.014** | **0.016** | 0.291 | 0.958 | **0.002** | **0.011** | 0.188 | 0.317 | 0.787 |  |  |  |
| AUTOPHAGY | | | | | | | | | |  |  |  |
| ATG7 | **0.002** | **0.014** | 0.299 | 0.958 | **0.002** | **0.011** | 0.188 | 0.436 | 0.787 |  |  |  |
| mTOR | **0.0005** | **0.014** | 0.291 | 0.958 | **0.002** | **0.011** | 0.188 | 0.981 | 0.787 |  |  |  |
| RAB5A | **0.002** | **0.014** | 0.291 | 0.958 | **0.002** | **0.011** | 0.188 | 0.436 | 0.787 |  |  |  |
| RAB7 | 0.115 | **0.028** | 0.584 | 0.958 | **0.054** | **0.019** | 0.867 | 0.558 | 0.787 |  |  |  |
| UPR | | | | | | | | | |  |  |  |
| IRE1α | **0.0005** | **0.013** | 0.291 | 0.958 | **0.088** | **0.000000002** | **0.001** | 0.440 | 0.787 |  |  |  |
| PROTEASOME 26S | 0.483 | 0.109 | 0.764 | 0.958 | 0.756 | 0.371 | 0.965 | 0.557 | 0.787 |  |  |  |

This table summarizes the *q* values obtained after false discovery rate (FDR) correction for all genes and proteins analyzed across the main statistical models. Values in bold indicate *q* < 0.05 after FDR correction.
